# Toward an Account of Intuitive Time

**DOI:** 10.1111/cogs.13166

**Published:** 2022-06-22

**Authors:** Ruth Lee, Jack Shardlow, Christoph Hoerl, Patrick A. O'Connor, Alison S. Fernandes, Teresa McCormack

**Affiliations:** ^1^ School of Psychology Queen's University Belfast; ^2^ Department of Philosophy University of Warwick; ^3^ Department of Philosophy Trinity College Dublin

**Keywords:** Temporal, Time, Metaphysics, Intuitive theories, Concepts, Constructs

## Abstract

People hold intuitive theories of the physical world, such as theories of matter, energy, and motion, in the sense that they have a coherent conceptual structure supporting a network of beliefs about the domain. It is not yet clear whether people can also be said to hold a shared intuitive theory of time. Yet, philosophical debates about the metaphysical nature of time often revolve around the idea that people hold one or more “common sense” assumptions about time: that there is an objective “now”; that the past, present, and future are fundamentally different in nature; and that time passes or flows. We empirically explored the question of whether people indeed share some or all of these assumptions by asking adults to what extent they agreed with a set of brief statements about time. Across two analyses, subsets of people's beliefs about time were found consistently to covary in ways that suggested stable underlying conceptual dimensions related to aspects of the “common sense” assumptions described by philosophers. However, distinct subsets of participants showed three mutually incompatible profiles of response, the most frequent of which did not closely match all of philosophers’ claims about common sense time. These exploratory studies provide a useful starting point in attempts to characterize intuitive theories of time.

## 1 Introduction

### 1.1 Intuitive theories and time

People seem to hold sets of both tacit and explicit beliefs about aspects of the physical and biological world, such as gravity, motion, life, and illness (Shtulman, 2017). These sets of beliefs about particular domains have been referred to as “theories” to reflect respects in which they resemble scientific theories: they form a coherent set, can be used to make inferences, and go beyond what is readily observable (Gerstenberg & Tenenbaum, 2017). However, they have also been qualified as “intuitive” (or “folk”) theories to reflect the fact that they are acquired in the absence of formal scientific education about the relevant domain.^1^

Do people also hold an *intuitive theory of time*? Although there is a large body of psychological research on, for example, the way people represent time spatially (e.g., Bender & Beller, 2014; Casasanto & Boroditsky, 2008; Tillman et al., 2018), on attitudes toward time (e.g., Caruso et al., 2008; Greene et al., 2021; Lee et al., 2020; Lee et al., 2022; Mello et al., 2013), and on how time is encoded in language (e.g., Boroditsky, 2011; Evans, 2013), there are only the beginnings of empirical research on intuitive beliefs about time. Moreover, what there is by way of research on this issue does not tell us whether people's intuitive beliefs about time can be said to form a theory, and, if they do, whether there is consistency across individuals as regards the elements of that theory.

Interestingly, something like the idea that people possess an intuitive theory of time already features in debates within philosophy about the metaphysical nature of time (see, e.g., Zimmerman, 2008). In the relevant philosophical contexts, the key debate has been about whether people's intuitive beliefs about time capture the metaphysical reality of time itself and, if not, what might explain why people hold those beliefs about time (see, e.g., Callender, 2017; Hoerl, 2014; Ismael, 2016).

What are the beliefs about time that philosophers have ascribed to people in this context? We take the following to describe three central assumptions that philosophers often take to be part of the common sense picture of time, with the caveat that this is not intended as an exhaustive description.

- **The Objective Now Assumption**. On this assumption, there is a moment in time that is objectively the present moment, rather than which moment is “now” being merely a matter of one's perspective in time. This assumption is typically also held to entail that the moment which is “now” is the same for everyone everywhere. It can be helpful to consider the contrast with space and the determinant of the referent of “here.” People do not assume that there is one objective “here,” but that what counts as “here” depends only on an individual person's perspective. By contrast, on the Objective Now Assumption, there is something about the present moment itself that makes it objectively “now.”
- **The Past–Present–Future Difference Assumption**. This is the belief that the past, the present, and the future are fundamentally different in nature. Again, a contrast with locations in space is helpful: people do not believe that the region of space they are located in differs fundamentally from any other region of space, or that regions of space to their left versus their right are fundamentally different in nature. By contrast, on the Past–Present–Future Difference Assumption, regions of time in the past, present, and future are conceived of as different from each other due to differences in the nature of the past, present, and future, or because the future *direction* of time is different in nature from the past *direction* of time. There are a variety of ways people are claimed to conceive of these differences, often associated with the idea that the past is “fixed,” whereas the future is “open,” or with the idea that things in the present are thought to be in some sense more “real” than those in the past and future. We will discuss these and other ideas in more detail below.
- **The Dynamicity Assumption**. In illustrating this assumption, it is once again useful to contrast time with space. Although people understand space as a domain in which objects can move (i.e., exhibit dynamic properties), they think about space itself as static. By contrast, on the Dynamicity Assumption, it is not just things in time that have dynamic properties, but time itself does so. Here too, there are are a variety of ways in which this idea has been spelled out. These include the idea that time “passes” or “flows” in a way that has no equivalent in space (i.e., that people have “Passage Beliefs”; see, e.g., Torrengo, 2017), and that it does so in a given direction (“Directionality Beliefs”; see, e.g., discussions in Savitt, 1995).

Many philosophers take one or more of these assumptions or beliefs to be central to people's common sense view of time (see, e.g., Callender, 2017; Miller, 2008; Zimmerman, 2008). In doing so, they are not claiming that people are necessarily metaconceptually aware of the beliefs that constitute this view. They seem to suggest, however, that these beliefs do constitute an intuitive theory: that, at least when prompted in the right way, people will acknowledge this set of commitments; that they concern properties people understand to be part of the nature of time; and that they constrain people's inferences. We will refer to this set of beliefs as “common sense time,” reflecting the idea that these beliefs, or some subset of them, form part of people's common sense picture of the world. Our approach in this paper is to investigate empiricially whether people do indeed typically share some or all of these beliefs, and whether the beliefs people hold about time amount to a theory in any principled sense.

### 1.2 The common sense versus the scientific picture of time

Part of the reason why philosophers have been discussing the beliefs constituting common sense time is that the view of the nature of time emerging from modern physics (henceforth, “scientific time”) is often held to be inconsistent with common sense time. As the theoretical physicist Carlo Rovelli (2019, p. 5) puts it: “We conventionally think of time as something simple and fundamental that flows uniformly, independently from everything else, from the past to the future, measured by clocks and watches. In the course of time, the events of the universe succeed each other in an orderly way: pasts, presents, futures. The past is fixed, the future open… And yet all of this has turned out to be false. One after another, the characteristic features of time have proved to be approximations, mistakes determined by our perspective, just like the flatness of the Earth or the revolving of the sun.” Thus, if Rovelli is correct, then the common sense picture of time is profoundly mistaken.

While there are differences between competing contemporary scientific theories of time, they tend to have in common the implication that scientific time is in conflict with common sense time. In particular, scientific time does not include the concept of an objective “now” that exists independently of a given person's perspective in time, meaning that the Objective Now Assumption is incorrect. The idea of an objective present is taken to be ruled out by the relativity of simultaneity––there being no absolute fact as to whether two spatially separated events are simultaneous or not (see, e.g., Callender, 2017, chap. 2). Instead, what people take to be “the present time”––and thus what they take to be past and future––is said to be merely a matter of their own perspective, as creatures whose own thoughts unfold in time. In that sense, “now” is in fact like “here.”

Note that, if true, this entails that at least some ways of characterizing time that have been associated with the Dynamicity Assumption are also ruled out. In particular, if there is no objective fact of the matter as to which moment of time is present, there is also no room for the idea of an objectively “moving now”––a change in which particular moment is the present moment in time––which is how the idea of the passage of time has sometimes been conceptualized. As such, while scientific time might allow for there to be a temporal order among events––certain events happen before or after certain others––time cannot itself be dynamic in the way some conceptions of the passage of time envisage.

Furthermore, if there is no objective, perspective‐independent fact of the matter as to *which* regions of time constitute the past, present, and future, these can also not be thought of as objectively distinct regions of time that differ in their fundamental natures. So, on at least some ways of conceiving of the Past–Present–Future Difference Assumption, this assumption also conflicts with scientific time.

Thus, if people possess an intuitive theory of time that broadly corresponds to what we are calling common sense time, and that embodies certain common ways of conceiving of the Objective Now, Past–Present–Future Difference, and Dynamicity assumptions, then it is inconsistent in fundamental ways with the central tenets of scientific time.

### 1.3 The philosophical analysis of time

The ostensible conflict between common sense time and scientific time is important in philosophy in the context of debates about the metaphysics of time, that is, debates about the nature of time itself. If common sense time conflicts with scientific time, this raises the question as to whether people's intuitive beliefs about time can reveal something about time itself that perhaps physics has not revealed (see, e.g., Markosian, 2004; Maudlin, 2002; Zimmerman, 2008), or whether people's intuitive beliefs about time are in fact fundamentally misguided (Callender, 2017; Price, 2011; Smart, 2008). Although it is well beyond the scope of the present paper to enter into this metaphysical debate, examining it briefly is useful because, even on the assumption that scientific time conflicts with common sense time, there are different ways of understanding what that conflict comes to.

Broadly speaking, we can distinguish between three different philosophical views about the metaphysics of time that different theorists have argued for (which we will return to later). These descriptions are not intended to be exhaustive. First, according to the view often referred to as Presentism, only things in the present exist; things in the past and future do not (see, e.g., Ingram, 2019; Markosian, 2004; Zimmerman, 2008). Alternatively, some philosophers hold the view that things in the past and the present exist but not things in the future—this is often known as the Growing Block view of time (see, e.g., Broad, 1923; Tooley, 1997). Finally, there is the view that things in the past, present, and future are all equal in terms of existence, a view known as Eternalism (see, e.g., Le Poidevin, 1991; Smart, 2008); a version of this claim is known as the Block Universe view of time, and often thought to be most consistent with the scientific picture of time (see, e.g., Mellor 1998; Price, 1997).

How do these metaphysical views fit with claims about common sense time? Some theorists think that Presentism (the view that only present things exist) is a feature of common sense time. Zimmerman (2008, p. 321), for example, argues that “it is simply part of commonsense that the past and future are less real than the present; that the difference between events and things that exist at present, and ones that do not, goes much deeper than the difference between events and things near where I am and ones that are spatially far away…”. Note that, on this suggestion, important metaphysical commitments about what is “real” or “not real” are built into common sense time. However, other philosophers discussing common sense beliefs about time, by contrast, tend to appeal less to beliefs about reality or existence, but instead to things like the idea that time passes or flows (what we have referred to as Passage Beliefs); that it does so in a given direction (what we have referred to as Directionality Beliefs); or the idea that the past is “fixed” and the future is “open” (one way of conceiving of the Past–Present–Future Difference Assumption: see, e.g., Forrest, 2004; Miller, 2008).

This variability in philosophers’ focus when describing the supposed features of common sense time suggests that caution is necessary in assuming that intuitive thought about time has been correctly characterized within that discipline. Worries that philosophers’ own theories may influence their characterizations of people's common sense beliefs have already been raised with respect to other domains (Knobe & Nichols, 2008; Nadelhoffer & Nahmias, 2007). Thus, although the characterizations given by philosophers provide a very rich starting point when considering the nature of people's beliefs about time, establishing what those beliefs are is an empirical matter that requires psychological research.

### 1.4 Existing findings

There is as yet very limited empirical evidence regarding the particular common sense beliefs about time that people typically hold, and no studies of which we are aware have addressed whether these beliefs cohere in the manner of an intuitive theory.

Shardlow et al. (2021) investigated how people understand what it means for time to pass in the context of a study of people's beliefs about their own experience of time. The authors asked directly for people's subjective reports of their experience––that is, whether they thought that they could feel or see time passing––and examined the relation between people's responses and their understanding of what time's passage in fact is. While a majority of participants did claim to feel and see time passing, and also understood talk of time passing in a way which broadly conflicts with scientific time, a minority did not. In a similar vein, Hershfield and Maglio (2020) investigated people's judgments about the duration of the present––that is, when the present ends and when the future begins––in order to test the hypothesis that such judgments are linked to future‐oriented decision making. Like Shardlow et al., these authors were primarily probing people's beliefs about their own experiences of time. Participants were explicitly told that, while they could answer a question about the duration of the present in objective terms, the researchers were more interested in what they *feel*, and the participants were thus instructed to answer the question: “When do you feel like the present ends?” Hershfield and Maglio found that although many people think that the present ends immediately, there is considerable variability.

By contrast with those two studies, our central concern in the current study was not with how people experience time and their beliefs about those experiences, but with people's beliefs about time itself and the degree to which these beliefs cohere in the manner of a theory. The most relevant existing studies are those of Latham et al. (2020, 2021a, 2021b; Latham & Miller, 2021), who investigated people's beliefs about time using vignettes. Latham et al.’s (2021a) participants read contrasting descriptions of universes with different temporal properties, each corresponding to a different philosophical model of the metaphysics of time. Their task was to judge which universe most resembles our own. Latham et al. distinguished between two classes of model, with their universe descriptions being categorized as “dynamical” or “nondynamical.” What they mean by *dynamical* here is a particular way of conceiving of the Dynamicity Assumption, one committed to the Objective Now Assumption, embodied in a general theoretical approach taken by some groups of philosophers: “Dynamists hold that events are ordered in terms of whether they are objectively past, present or future; the location of events within that ordering is dynamic in that a set of events, *E*, is future, will be present, and will then become past. According to dynamists time flows by virtue of a set of events being objectively present, and which sets of events that is, changing. Dynamists take tensed thought and talk to pick out genuinely dynamical… properties” (p. 4252).

Latham et al. demonstrated that the majority (∼ 70%) of people chose a model characterized as dynamical, but also that a substantial minority chose a nondynamical model. In a follow‐up study, Latham et al. (2020) explored whether people represent time as *essentially* dynamical—whether something has to be dynamical in order to be time. They modified some of their vignettes to avoid reference to time as much as possible, and then asked participants to judge whether there was such a thing as “time” in universes that were described in either dynamical or nondynamical terms. In this follow‐up, the authors found that, on the whole, participants tended to judge that there is time in every scenario (so, e.g., even if a participant had selected a dynamical description as the best description of our actual universe, they typically judged that there was time in a universe that was described as nondynamical). Latham et al.’s general conclusion was that people do not appear to believe that time is essentially dynamical. Extending this line of research, Latham et al. (2021b) found that while most participants did not judge a specific aspect of dynamicity––the directionality of time––to be necessary for there to be time in a world, the presence of directionality bolstered judgments that there is time in a world. However, as before, participants rarely judged that there is no time in a given world, even one devoid of directionality; Latham et al. (2021b) similarly found that people were ready to judge that there is time even in a world in which temporal phenomenology is explicitly described as being illusory because there is no genuine causation or change.

Latham et al.’s (2020, 2021a, 2021b; Latham & Miller, 2021) studies provide an important first step in exploring common sense time. However, because they were attempting to describe whether intuitive theories matched some existing philosophical theories about time, their method involved vignettes that were very complex. Moreover, this method involved bundling together particular sets of beliefs about time. The vignettes in Latham et al.’s (2021a) study touched on all three of the assumptions we listed in Section 1.3, as well as a number of other features. So, for example, the descriptions all began with a summary of what is real or exists in that universe (e.g., “Imagine a universe in which the only objects and events that exist are those in the present moment…” was a key part of the description of a philosophical model corresponding to Presentism and classified by Latham et al. as dynamical). Furthermore, in some instances, the descriptions explicitly specified whether something like the Objective Now Assumption holds in that universe (e.g., “No set of events is special. Every event is present from the perspective of those located at it, just as every location is ‘here’ from the perspective of those located at it” was a part of a description of another model that was not compatible with the Objective Now Assumption and was classified as nondynamical). The use of bundled beliefs is not surprising because philosophers have carefully considered whether holding one particular belief means that one should also hold other types of related beliefs (e.g., whether believing that time passes means, to be theoretically consistent, that one should also hold the Objective Now Assumption or vice‐versa). Importantly, it is not clear to what extent people's actual common sense beliefs about of time do bundle together in such a theory‐like way.

### 1.5 The current study

In the present study, we examined whether people possess a shared set of beliefs about time and, if they do, whether these beliefs cohere in a way that resembles an intuitive theory, or whether they are disparate and variously related. Given the potential complexity of people's constructs of time, and the limited relevant empirical work to date on these questions, this project must necessarily begin as highly exploratory. In the current study, rather than using vignettes, adults were asked directly to what extent they agreed with a set of brief statements about time, such as “Time has a direction.” We included statements that were (broadly) categorized as connected with the Objective Now, Past–Present–Future Difference, and Dynamicity Assumptions described above; more detail is provided in Section 2.2.1.

After examining whether, as a group, participants tended to agree or disagree with this set of statements, we then explored the underlying structure of participants’ beliefs about time by looking for patterns of covariation across levels of agreement with these statements. If beliefs about time are largely independent of one another, they may (e.g.) simply reflect people's familiarity with generally accepted metaphors for time. If, however, patterns of beliefs reliably covary, we might reasonably infer that they reflect underlying, qualitatively distinct constructs and conclude that people think about time in broadly metaphysically loaded terms, holding something like an intuitive theory of time. By looking for patterns in these constructs, we might also identify subpopulations who hold somewhat different intuitive theories of time; indeed, study 2 directly addressed this issue of subpopulations.

## 2 Study 1

In study 1, we presented participants with a large number of statements about time and explored their responses in relation to the assumptions identified above. Rather than simply examining whether participants agreed or disagreed with specific statements, our analyses also used the technique of exploratory factor analysis (EFA) in order to look for evidence of underlying dimensions in patterns of beliefs: that is, latent belief constructs. In factor analysis, indicator variables are assumed to be a function of one or more underlying factors (plus error), meaning that variation in the latent construct leads to variation in its measures. EFA computes how much of the association between variables can be explained by one or more such factors, accounting for as much covariation as possible (common variance) until an acceptably small amount of covariation not accounted for by the factors (unique variance) remains. Variables are said to “load onto” factors: that is, to covary in a way that allows the researcher to identify the potential latent construct that might explain the covariation. For instance, in clinical studies, certain associated variables representing patterns of thought and behavior might be interpretable as the latent construct “general depression,” whereas another set of associated variables might be said to represent the latent construct “agitation and anxiety” (Li et al., 2014).

Ethical approval for this and the second study was received from the research ethics committee of the first author's institution.

### 2.1 Method

#### 2.1.1 Materials

Data collection took place online using the Qualtrics platform, and participants completed the questionnaire on desktop, laptop, or mobile devices.

#### 2.1.2 Participants

Two hundred and four participants (*M* = 31.0 years, *SD* = 11.76, range: years, 96 males) were recruited from the Prolific online subject pool (Peer et al., 2017) and received compensation of £2.66 UK pounds. Seventeen participants (*M* = 20.6 years, *SD* = 3.69, range: 18–74 years, three males) were recruited through an undergraduate research pool and received course credit. The full sample thus comprised 221 participants (*M* = 30.19 years, *SD* = 11.68, range: 18–74 years, 99 males). All participants stated that they were fluent in English.

#### 2.1.3 Design and procedure

The study comprised 33 pairs of Beliefs About Time statements, each consisting of a statement and its converse, for a total of 66 statements. Pilot work conducted prior to this study included additional statements related to the Objective Now assumption, which were dropped due to concerns that participants found them difficult to interpret. All participants first provided informed consent, and then their age and gender. They then responded to the Beliefs About Time statements. Participants were randomly allocated to one of four counterbalanced conditions, each of which presented the Beliefs About Time statements in a different quasi‐randomized order. No question appeared within two questions of its converse. Finally, participants indicated their level of education, and responded to the statement “Within the last three years I have read, watched, or listened to something about how scientists think time works.” Response options were “Never,” “Once,” “Two or three times,” and “More than two or three times.” They also responded to four additional statements related to time and answered a further demographics question, which were not the focus of this study and will not be discussed further here. Participants were not able to skip any questions, although they were able to select a “Don't Know” option and then to choose a reason for selecting that option (described below).

##### 2.1.3.1 Beliefs About Time statements

**Fig. 1:**
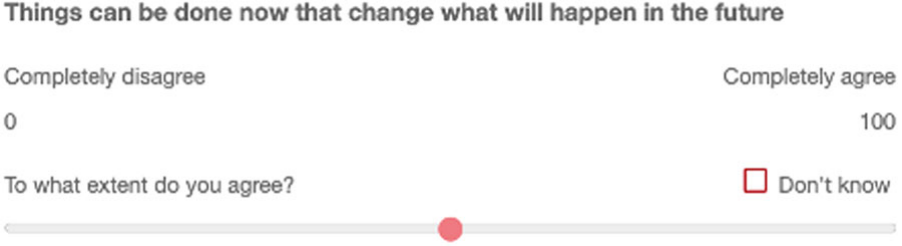
Example of a Beliefs About Time statement.

Beliefs About Time statements are listed by assumption in Appendix A. Participants saw a scale running from 0 to 100, where 100 represented “completely agree” and 0 represented “completely disagree.” A red dot was situated at the midpoint of the scale (50). The accompanying text asked participants to move the dot along a sliding scale to indicate the number that best reflected the degree to which they agreed or disagreed with a statement (Fig. 1). As online participants moved the dot, they saw a number reflecting its current location on the scale. Participants were also informed of the “Don't Know” option. Each Belief statement was presented twice, once in the positive (e.g., “We have some control now over what will happen in the future”), and once in the negative (e.g., “We have no control now over what will happen in the future”). Participants also responded to an additional initial practice statement (see Supplementary Materials). If participants selected the “Don't Know” option in response to the practice statement or one of the Belief statements, they were presented with an additional question: “Which is closer to what you were thinking when you selected this option?,” and had to choose between “I don't personally know to what extent the statement is true or untrue,” “I don't think it is possible to know to what extent the statement is true or untrue,” and “I don't understand the question.”

#### 2.1.4 Data scoring

Appendix B reports means, 95% confidence intervals, and Spearman–Brown split‐half coefficients describing the reliability of pairs of positively and negatively worded versions for Beliefs About Time statements.

Pairs of statements (i.e., a statement plus its converse) demonstrated variable reliability (split‐half coefficients between −0.454 and 0.764). Six pairs of statements were excluded from the analysis due to low reliability (see Supplementary Materials). This left 60 Beliefs About Time statements, 27 of which formed pairs. Following the exclusions, Spearman–Brown split‐half coefficients ranged between 0.17 and 0.76. As reliability was variable, we did not collapse responses to pairs of positively and negatively worded statements prior to analysis.

For the purposes of initial descriptive analysis, dichotomized scores were calculated for Beliefs About Time statements by categorizing values over 50 as agreement with the statement, values below 50 as disagreement with the statement, and values of 50 (the midpoint) as missing. Dichotomized scores for Beliefs About Time statements are reported in Appendix A. All but five of the 60 Beliefs About Time variables (93.3%) included “Don't Know” responses. These were excluded from descriptive analyses, yielding slightly different *n*s for each variable. The proportion of participants who chose any one of the three “Don't Know” options for Beliefs About Time statements ranged from 0% to 10.9% on each variable, with a mean of 3.4%.

Participants’ responses on one of each pair of variables were reverse scored prior to data imputation and analysis by subtracting the value of each response from 100. Note that Appendix B presents mean values and 95% confidence intervals *prior to* reverse scoring, and split‐half values *following* reverse scoring. Missing data resulting from “Don't Know” responses were imputed using a single predictive mean matching imputation (see Supplementary Materials).

### 2.2 Results

We first examined the extent and variability of participants’ level of agreement with the Beliefs About Time statements. We then used EFA to explore the pattern of participants’ endorsement of the Beliefs About Time statements for the presence of underlying latent constructs, using the dataset containing imputed values.

#### 2.2.1 Descriptive statistics

Because of the size and complexity of the data set and given that not all statements were subsequently associated with underlying constructs (described in Subsection 2.2.2), here we will focus on simply commenting on the pattern of data under dichotomized scoring. Appendix A shows the proportion of participants who endorsed or rejected each statement (i.e., gave a score of ≥ 51 or ≤ 49); in the table, pairs of statements (a statement plus its converse) are presented on adjacent lines. The data in the table are structured according to which of the three assumptions (outlined in 1.1) the relevant statement is most clearly related to. In what follows, we will designate statements for which approximately 90% of participants agreed (either in terms of endorsement or rejection) as showing *very highly* consistent agreement, approximately 80% agreement as showing *highly* consistent agreement, and approximately 70% agreement as showing *moderately* consistent agreement.

(i) **Objective Now Assumption**. The statements most closely related to this assumption were: “When an event turns from being future to being present to being past, something about the event itself changes” and “Whether an event is past, present, or future is a fact about that event itself rather than just our perspective on it,” plus the reverse of the statements. As can be seen from Appendix A, although in all instances the majority of people made judgments consistent with the Objective Now Assumption, levels of consistent agreement for all of these statements were relatively low (typically ∼55–60%). In that respect, these data do not provide good evidence in favor of the idea that the Objective Now Assumption is part of the common sense view of time; we return to this in the Discussion of study 1.
(ii) Past–Present–Future Difference Assumption. We can consider the statements relevant to this assumption as falling under two broad subcategories: (a) statements relating to what is “real” or the “fundamental nature” of the time periods (labeled Reality and Fundamental Nature in Appendix A) and (b) statements relating to the ideas that the past is somehow more fixed and knowable by contrast to the future which is more open and less certain (labeled Control and Certainty in Appendix A).
(a) *Reality/Fundamental Nature*. With regard to these statements, we were interested in whether participants showed evidence of holding what philosophers refer to as “Presentist” beliefs, that is, that only things in the present are real (Markosian, 2004; Zimmerman, 2008). We were also interested in whether participants thought that the future and the past differed in terms of how real they are; this is because there is also an alternative philosophical position, according to which only things in the past and the present are real (the so‐called Growing Block model of time; Broad, 1923; Tooley, 1997).

With regard to the first issue, even though there was moderately consistent agreement to statements concerning whether the past or the future are “fundamentally different in nature,” there was no evidence that people typically thought of things in the past or the future as “not real.” In fact, when asked whether they agreed with the statement “Things are only real if they are in the present,” there was moderately consistent agreement that this statement was false (∼73%). Indeed, the strongest level of consistent agreement to the statements in this category was to “Things in the past are real, even if they are not in the present,” which over 90% agreed with. Thus, these data provide no positive support for the idea that the majority of people hold Presentist views.

We then turned to the second issue: whether there is any evidence that people believe that the past and present are real, but the future is not. While there was no evidence that people typically judged that things in the future are not real, nevertheless, a series of McNemar's tests (for simplicity, conducted only on positively worded statements) indicated that people were more reluctant to say that things in the future are real than to say that things in the past are real. Specifically, people were significantly more likely to agree that things in the past are real (90.83%) than to agree that things in the future are real (67.48%), *p* < .001. Consistent with this, people were also significantly more likely to agree that things in the past are real in the same way as things in the present (73.52%) than to agree with the equivalent statement about the future (46.76%), *p* < .001. Thus, although the data do not suggest that people typically believe only things in the present are real, there was some evidence that they were more likely to say that things in the past are real compared to things in the future.

(a) *Control and Certainty*. All bar one of the statements in this subcategory showed at least moderately consistent agreement and many showed highly or very highly consistent agreement. The general pattern of responses indicated that most people think of the past as different from the future in terms of controllability, possibility of change, and knowledge. Two McNemar's tests on positively worded statements confirmed this: people were significantly more likely to agree that we have some control now over what will happen in the future (94.39%) versus the past (30.59%), *p* < .001, and significantly more likely to agree that things can be done now that change what will happen in the future (97.70%) versus the past (24.20%), *p* < .001. The great majority of participants agreed with the statement that more can be known about the past than about the future (90.83%). Thus, the findings suggest that the majority of people consider the past to differ from the future in a number of key respects.
(b) **Dynamicity Assumption**. We can again think of there being two subcategories: (a) Directionality and (b) beliefs about Time Passing/Flowing.
(c) *Directionality*. All statements in this subcategory showed at least moderately consistent levels of agreement, with the higher levels of consistent agreement to the statements “Time flows forward” (89.47%) and “Time has a direction” (82.44%). Taken as a whole, the responses to these questions suggest that people do conceptualize time as directional in some sense.
(d) *Time Passing/Flowing*. We set aside the questions regarding the meaning of the statement “time flows” due to their negative reliability across pairs (see Supplementary Materials). With regard to the other items, there were at best moderately consistent levels of agreement, observed only to the statements “We are moving in relation to time” and its converse, to “The present moves forward in time,” and to “Things in the past move away from us.” A McNemar test demonstrated that significantly more participants endorsed “We are moving in relation to time” (76.33%) than endorsed “Time is moving in relation to us” (64.04%), *p* = .004. Thus, although the majority of people seem to agree with the idea that time has directionality, this does not seem to translate into general agreement with statements that attempt to specify how best to describe time's flow.

#### 2.2.2 Exploratory factor analysis

Analyses were conducted using the package “psych” (Revelle, 2019) in R (R Core Team, 2018). We used principal axis factoring, which is robust to multivariate violations of normality (Fabrigar et al., 1999). For clarity and simplicity of interpretation, results were subsequently rotated in the space describing the relationship of each variable to multiple factors. We did not anticipate that the latent constructs underlying people's beliefs about time were likely to be entirely orthogonal. We, therefore, used oblique rotation (direct Oblimin), such that the rotated axes were not constrained to remain perpendicular and so factors were allowed to correlate. Data were found to be suitable for factor analysis based on their Kaiser–Meyer–Olkin value (KMO) of 0.7 (Tabachnick & Fidell, 2013) and by Bartlett's test of sphericity (*p* < .001). Model selection is described in the Supplementary Materials.

#### 2.2.3 Factor solution

A four‐factor solution was accepted. The internal consistency and discriminant validity of this solution are described in the Supplementary Materials. The final Cronbach's alpha exceeded 0.7 for each factor (Table 1).

**Table 1:** Factor loadings, Cronbach's alpha, and proportion of variance explained for each factor

	Factors			
	Open Future	Mutable Past	Presentism	Directionality
α (*SE*)	0.81 (0.77, 0.85)	0.81 (0.77, 0.85)	0.72 (0.66, 0.78)	0.74 (0.69, 0.80)
% variance explained (all variables)	0.15	0.13	0.11	0.10
% variance explained (within model)	0.31	0.27	0.22	0.20
Eigenvalue	4.3	1.83	1.37	0.94

The factors were named as follows:

Factor 1, Open Future: the statements that loaded on this factor addressed control of and agency over the future, its epistemological status, and the fundamental natures of the past and future.
Factor 2, Mutable Past: the statements that loaded onto this factor addressed control of and agency over the past.
Factor 3, Presentism: the statements that loaded onto this factor addressed the reality of the future and past as compared to the present.
Factor 4, Directionality: the statements that loaded onto this factor addressed the question of whether there was a direction to time, the nature of that direction, and whether any such direction is reversible.

It is important to emphasize that identification of these factors does not mean that there was typical agreement among people regarding the statements. For instance, as we have pointed out above, there was no evidence that the majority of people had beliefs consistent with Presentism. Rather, the factors indicate that responses on some statements (identified below) tended to cohere in interpretable ways.

#### 2.2.4 Properties of the four‐factor model

The four‐factor model is presented in Table 2, containing factor loadings and communalities, and its properties are presented in Table 1, containing Cronbach's alpha, eigenvalues, and the proportion of variance explained for each factor. Interfactor and interitem correlations are presented in the Supplementary Materials (Table S1). Two assumptions were represented: Past–Present–Future Difference, including the subcategories of Reality and Fundamental Nature and Control and Certainty; and Dynamicity, including the subcategory of Directionality, but not that of Time Passing/Flowing. The third assumption, Objective Now, was not represented. The cumulative proportion of variance explained by all four factors was 48%. While in the natural sciences, the expectation for a useful solution is often a minimum explained variance of 60%, given that self‐reported information about a rarely discussed concept (such as time) is often imprecise, a somewhat lower figure is considered satisfactory (Hair et al., 2006).^2^

**Table 2:** Factor loadings and communalities (h2) for the retained four‐factor model

Variable	Open Future	Mutable Past	Presentism	Directionality	h2
We can always have more knowledge about the past than we can about the future	0.48				0.42
The future is not yet settled	0.81				0.39
Things can be done now that change what will happen in the future	0.85				0.55
Nothing can be done now that changes what will happen in the future^a^	0.58				0.52
We have no control now over what will happen in the future^a^	0.47				0.39
The present and future are fundamentally different in nature	0.41				0.21
Not only can we change how we think or feel about the past, but we can also change what actually happened		0.64			0.54
Things can be done now that change what has happened in the past		0.76			0.57
We have some control now over what has happened in the past		0.73			0.60
We have no control now over what has happened in the past^a^		0.61			0.50
Things in the future are not real			0.57		0.33
Things in the future are real^a^			0.58		0.34
Things in the future are real in the same way as things in the present^a^			0.77		0.63
Things in the past and the future are just as real as things in the present^a^			0.62		0.41
Time only flows forward. It could never flow backward				0.78	0.63
It is not possible for time to reverse its direction				0.70	0.50
It could be possible for time to reverse its direction^a^				0.64	0.51
Time has a direction				0.44	0.21

*Note*. ^a^Reverse‐scored.

### 2.3 Discussion

Study 1 explored a number of features that philosophers have suggested may form part of people's common sense beliefs about time. We begin by considering the descriptive data before turning to the findings of the factor analysis.

#### 2.3.1 Descriptive data

First, although most people agreed with positive statements that attempted to capture the Objective Now Assumption, this agreement was weakly consistent; it was also inconsistent in that it did not extend to the negations of the statements. Moreover, none of the statements loaded on the factors identified in the factor analysis. Unfortunately, lack of strongly consistent agreement to relevant statements in study 1 could reflect either genuinely different intuitions relating to the Objective Now Assumption, or participants’ differing interpretations of the statements. The statements we used to test the assumption focused on the ideas that something about an event itself changes when it changes tense and that whether an event is past, present, or future is a fact about the event itself. It is possible that these descriptions are open to a variety of interpretations (e.g., people differ in terms of what kinds of things could be considered “a fact about the event itself”). Future studies examining the Objective Now assumption might explore different ways of expressing this assumption, either by clearly elaborating on the proposed difference between the way people conceptualize “here” and “now” or by describing it in terms of the idea that which time is “now” is the same for everyone.

Second, although people do seem to believe that the past, present, and future differ in important ways (the Past–Present–Future Difference Assumption), this does not seem to straightforwardly extend to the idea either that only things in the present are real (Presentism) or that only things in the past and present are real (compatible with the Growing Block approach). Nevertheless, we are reluctant to conclude that people think of things in the past, present, and future as being equal in terms of existence (Eternalism). This is because, first, there was moderately consistent agreement with statements describing things in the past/future as being real but “not in the same way as the present,” and also with statements that the past/future are “fundamentally different in nature” from the present. Second, people were significantly more likely to agree to statements concerned with the reality of the past versus the reality of the future. The combination of these two findings is interesting and suggests that although the majority of people do not have clear shared ontological intuitions that correspond to either Presentism or the Growing Block view, nevertheless, they do think of past, present, and future as differing in some basic and important ways. In study 2, we returned to this issue by examining whether there are subpopulations of people who have beliefs that seem to map on to different views of reality and time espoused by different philosophers.

What is clearer from the data from study 1 is that there are other, perhaps more easily expressed, ways in which the majority of people think of the past and future as differing from each other. The future was judged as more controllable, easier to change, and less knowable than the past. In this respect, the beliefs of the majority of people resemble those ascribed to them by philosophers who consider common sense time to adhere to the idea that the past is somehow “fixed” by comparison to the “open” future (Miller, 2008; Forrest, 2004). Note that in designing the statements relating to these ideas, we were concerned that people might consider the past as not yet fixed in terms of people's attitudes or emotions toward it and as a result specifically included a statement “Not only can we change how we think or feel about the past, but we can also change what actually happened.” Most people disagreed with this statement, but, nevertheless, around 17% of people judged it to be true. As we describe below, in study 2, we explore whether there is an interesting subset of people who consistently judge the past not to be fixed.

Turning to the Dynamicity Assumption, people generally agreed that time has a direction, but there was less agreement about particular ways of describing the flow of time. Between 80% and 90% of participants agreed that time has a direction and that it flows forward; however, agreement with specific statements about the movement of things or points in time itself was much less consistent. One speculative possibility, which may reconcile these two sets of findings, is that people interpreted questions about time possessing a direction (forward flowing) primarily in terms of causation (or controllability of things in the future by comparison to the past), rather than in terms of any particular spatial metaphor about the movement of time or temporal frame of reference. By contrast, it could be that whether one agrees with the other statements (e.g., “Things pass from future to present to past” or “Time is moving in relation to us”) depends on the particular spatial metaphor or temporal frame of reference currently being adopted. Such an idea would be compatible with claims in the psycholinguistic literature that such metaphors or frames of reference are both variable and malleable (Bylund et al., 2020; de la Fuente et al., 2014; Núñez & Cooperrider, 2013).

#### 2.3.2 Factor analysis

The findings of the factor analysis were valuable, because they demonstrated that subsets of people's beliefs about time covary in ways that are theoretically interpretable. Thus, the results are compatible with the idea that people operate with something akin to an intuitive theory of time. The analysis yielded underlying dimensions of Open Future, Mutable Past, Presentism, and Directionality, suggesting that all of these dimensions are involved in structuring people's intuitive theory of time. These four dimensions relate to aspects of common sense time that have previously been proposed by philosophers: Open Future and (the denial of) Mutable Past are both associated with the Past–Present–Future Difference Assumption; Presentism is considered by some philosophers to be a consequence of the Objective Now and Past–Present–Future Difference Assumptions; and Directionality is, for many philosophers, an important feature of the Dynamicity Assumption.

As they stand, though, the findings of the factor analysis leave important issues unresolved. First, the model generated by study 1 arose from an entirely exploratory data process, meaning that it is important to demonstrate that it can be replicated in a study with a different sample of adults using only the same items that loaded on the factors. Second, even if the factor analysis successfully identified a shared set of dimensions that structure people's beliefs about time, it is possible that people, nevertheless, differ from each other in the extent to which they endorse (or reject) these features: that is, their beliefs along these dimensions may vary. As mentioned in the previous subsection, for example, it could be that there are some individuals who consistently make judgments that indicate that the past is fixed and immutable (indeed, this appears to be the majority), but another subset that makes judgments that indicate that they think of the past as something that is not fixed. Indeed, the latter subset might also differ in terms of their judgments about (e.g.) the directionality of time.

In study 2, we addressed these issues. We reran a version of the study using a questionnaire with only those items that had been identified in the factor analysis as loading on the dimensions and then used confirmatory factor analysis (CFA) to examine whether the factors identified in study 1 are robust. Subsequently, we used latent profile analysis (LPA) to investigate whether there are distinct subsets of participants with particular profiles of responses across the items. If the extent to which participants endorse the dimensions expressed in the four‐factor model is patterned in mutually exclusive profiles, this would suggest that we can potentially identify groups of people who hold distinct intuitive theories of time.

## 3 Study 2

### 3.1 Method

#### 3.1.1 Materials

Data collection took place online using the Qualtrics platform, and participants completed the questionnaire on desktop, laptop, or mobile devices.

#### 3.1.2 Participants

Two hundred and fifty‐one participants (*M* = 28.8 years, *SD* = 9.89, range: 18–76 years, 136 males) were recruited from the Prolific online subject pool (Peer et al., 2017), and received compensation of £1 UK pound. All participants stated that they were fluent in English.

#### 3.1.3 Design and procedure

All participants first provided informed consent, and then their age and gender. They then responded to the 18 Beliefs About Time statements (Table 2). Participants were randomly allocated to one of four counterbalanced conditions, each of which presented the Beliefs About Time statements in a different quasi‐randomized order, in which no question appeared within two questions of its converse. Participants next responded to the four additional statements related to time and the additional demographics question asked in study 1 (which were again not the focus of the current study and will not be discussed here), indicated their level of education, and responded to the question regarding exposure to reading, watching, or listening to material about how scientists think time works. As in study 1, participants were not able to skip any questions, although they were able to select a “Don't Know” option and then to choose one of three reasons for selecting that option.

#### 3.1.4 Data scoring and descriptive statistics

Appendix B reports means, 95% confidence intervals, and (in the case of six Beliefs About Time statements forming one half of a pair comprising a statement and its converse) Spearman–Brown split‐half coefficients for Beliefs About Time statements. The overall reliability of the questionnaire was adequate (Cronbach's α = 0.521). The three pairs of statements demonstrated variable reliability (split‐half coefficients between 0.414 and 0.813).

For the purposes of descriptive analysis, as in study 1, dichotomized scores were calculated for Beliefs About Time statements, as reported in Appendix C. All 18 Beliefs About Time variables included “Don't Know” responses. These were again excluded from descriptive analyses, yielding slightly different *n*s for each variable. The proportion of participants who chose any one of the three “Don't Know” options for Beliefs About Time statements ranged from 0% to 6.4% on each variable, with a mean of 1.16%. Inspection of the table in Appendix D indicates that the level of consistent agreement for each statement was very similar to that observed in study 1; for the majority of statements, the difference between study 1 and study 2 in the percentage of participants agreeing or disagreeing with a statement was within 1–5%, and the largest difference between studies in agreement or disagreement was approximately 14%. In this respect, these findings amount to a good replication of the findings of study 1.

### 3.2 Confirmatory factor analysis

Where Beliefs About Time variables constituted a pair comprising a statement and its converse (six statements; three pairs), one statement from each pair was again reverse scored prior to data imputation. Missing data were imputed using the procedure outlined in study 1 (Section 2.1). We first examined the adequacy of the fit of the four‐factor structure that emerged from the EFA performed in study 1 to the new data. Data were analyzed using the lavaan package (Rosseel, 2012) in R (R Core Team, 2018). All participants were included in the analysis. None of the indicators showed evidence of multicollinearity: all variance inflation factor scores were below the cutoff of 5, and all of the Tolerance scores were above the cutoff of 0.2 (Hanna & Dempster, 2013). Finally, the factorability of the data was confirmed by a KMO value of 0.719 and a significant Bartlett's test of sphericity (*p* < .001).

#### 3.2.1 Factor score estimation

Factor scores indicate a participant's relative standing on a latent factor (DiStefano et al., 2009). Thurstone factor scores are presented in Table 3. Refined factor scores, such as Thurstone scores, have a mean of 0 and produce approximately standardized estimates of the common variance of a factor by weighting the contribution of each variable according to a regression coefficient. These scores were used in subsequent analyses. For Open Future, Presentism, and Directionality, the higher the factor score, the stronger the agreement with the assumptions suggested by philosophers to be part of common sense time; for Mutable Past, the higher the factor score, the weaker the agreement with these assumptions.

**Table 3:** Descriptive statistics and interfactor correlations for the factor scores extracted from the CFA (*n* = 251)

				Correlations			
Factor	Mean	SD	[Min, Max]	(1)	(2)	(3)	(4)
(1) Open Future	0.00	0.94	−4.50, .88	–			
(2) Mutable Past	0.00	0.84	−1.14, 2.65	−.52**	–		
(3) Presentism	0.00	0.93	−1.46, 2.36	−.19**	−.13*	–	
(4) Directionality	0.00	0.93	−2.59, 1.02	.28**	−.58***	.18**	–

* *p* < .05; ** *p* < .01; *** *p* < .001.

#### 3.2.2 Model fit

As most of the data were non‐normally distributed, we employed the robust maximum likelihood estimation method, which provides robust standard errors and a robust chi‐square statistic (Satorra–Bentler correction: Bentler, 1995) to correct for non‐normality in CFA (Brown, 2006). Further information on goodness of fit, absolute fit, comparative fit, and parsimony correction indices is provided in the Supplementary Materials and is summarized in Tables 3 and 4. Taken together, the absolute and comparative fit indices and the parsimony correction indices indicated that the data were a good fit for the proposed four‐factor model concerning people's beliefs about time.

**Table 4:** Goodness‐of‐fit statistics for the four‐factor model

	χ2	df	*p*	χ2/df	CFI	TLI	RMSEA [90% CI]	SRMR
Model	188.64	129	< .001	1.46	0.94	0.93	0.05 [0.031, 0.061]	0.06

**Fig. 2:**
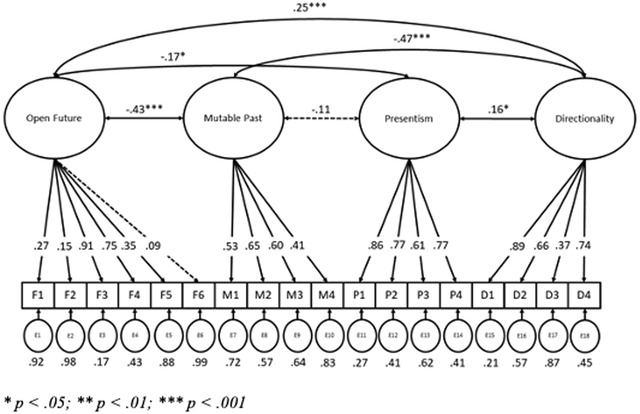
Path diagram with factor loadings, error variances, and factor covariances for the four‐factor Beliefs About Time model (nonsignificant paths are indicated by dotted lines). Statements are listed in full by item number (F1, M1, etc.) in Appendix D. * p < .05; ** p < .01; *** p < .001.

Fig. 2 shows the path model for the four‐factor model of beliefs about time. Unstandardized path coefficients are included in Appendix E, while correlations between each of the indicators are shown in Appendix F. The factors were all significantly correlated, with the exception of Mutable Past and Presentism. Furthermore, all of the items had factor loadings of above 0.3 onto their respective factors, with the exception of three items loading onto the factor Open Future (“We can always have more knowledge about the past than we can about the future”; “The future is not settled”; “The present and future are fundamentally different in nature”).

**Summary** The confirmatory factor analysis validated the patterns of covariation in subsets of beliefs about time seen in study 1 in a new sample. The underlying dimensions initially identified by the EFA were a good fit for the new data, providing evidence that the individual beliefs from which the factors were derived are indeed reflective of four distinct underlying dimensions: Open Future, Mutable Past, Presentism, and Directionality.

### 3.3 Latent profile analysis

We now turn to the question of whether particular patterns of beliefs about time are displayed by latent subpopulations. To address this question, we subjected the weighted Thurstone factor scores generated by the CFA to an LPA, performed using the tidyLPA package in R (Rosenberg et al., 2018), utilizing the maximum likelihood estimation method. LPA searches for subtypes of people who exhibit similar patterns of responses. Here, we use it to capture heterogeneity in the extent to which people endorsed the latent beliefs about time represented by the four factors identified in study 1: Open Future, Mutable Past, Presentism, and Directionality.

#### 3.3.1 Model specification and selection

The tidyLPA package was used to determine the best profile solution for the data. Details of model specification and selection are given in the Supplementary Materials.

#### 3.3.2 Results

**Fig. 3:**
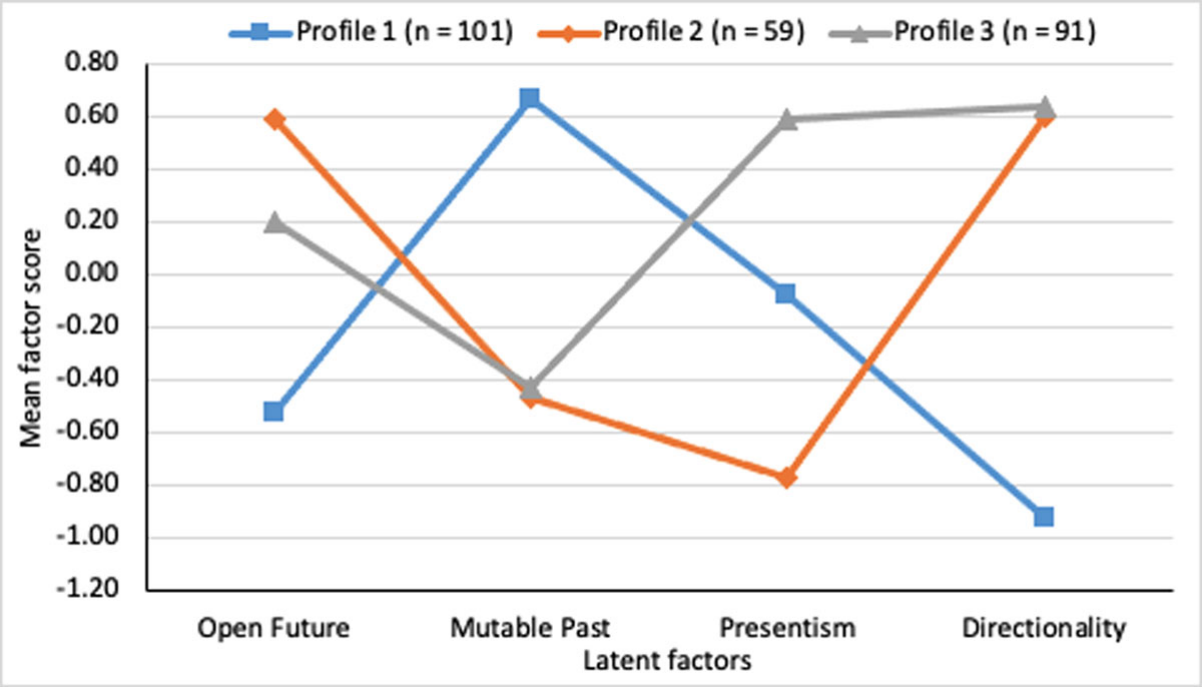
Line graph showing the three‐profile solution. Lines represent weighted Thurstone factor score means, indicating the relative standing of participants on each factor.

**Fig. 4:**
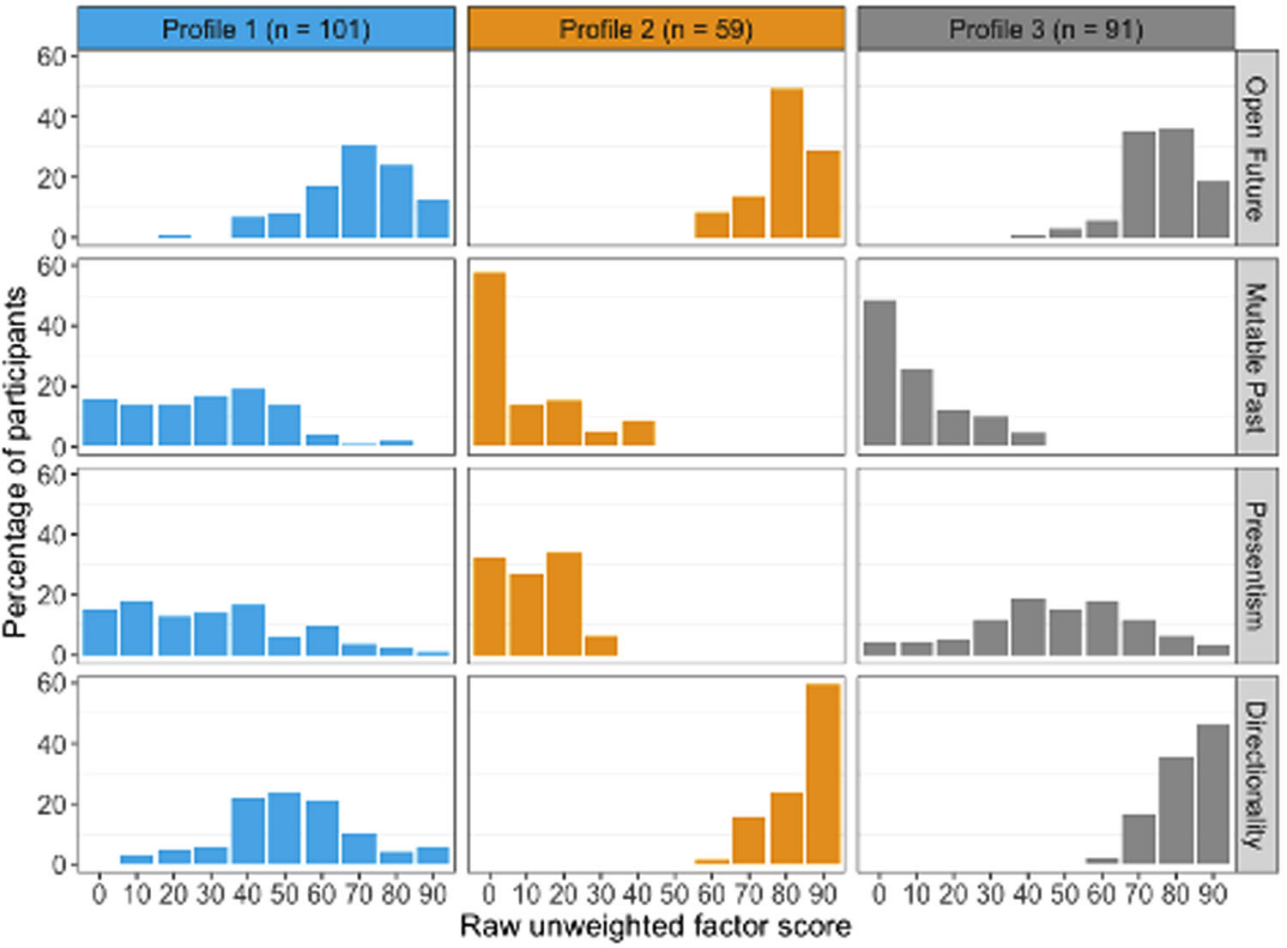
Histograms presenting the distribution of raw, unweighted factor scores within each profile for Open Future, Mutable Past, Presentism, and Directionality. Unweighted factor scores reflect participant responses on the original scale of measurement (0–100).

Results are illustrated below. First, the *relative* level of endorsement given by members of each profile to the constructs represented by each factor (using the weighted Thurstone factor scores) is illustrated in Fig. 3. This figure is helpful in displaying the ways in which the different profiles showed particular patterns across each of the four factors, but does not itself provide information about what the *actual* distribution of responses to the questions looked like. Thus, for illustrative purposes, we also calculated raw, unweighted factor scores for each of the three profiles in order to examine participant responses on the original scale of 0–100. The distribution of these scores for each factor is shown separately for each profile in Fig. 4; factors are listed to the right vertically and profile numbers listed at the top horizontally.

#### 3.3.3 Inspection and analysis of the profile data

Inspection of Fig. 3 suggests that profiles 2 and 3 shared more similarities with each other than with profile 1; profiles 2 and 3 only showed marked differences in terms of relative level of agreement with Presentism, whereas profile 1 appeared to differ from the other two across all four factors. Inspection of Fig. 4 sheds further light on the patterns of *actual* levels of agreement (see also Table S2). First, the large majority of participants in profiles 2 and 3 show moderate to strong agreement that the future is open, that the past is not mutable, and that time has a direction, consistent with the way aspects of common sense time have been characterized by philosophers. However, none of the participants in profile 2 endorsed Presentism, even weakly, whereas responses were more variable with regard to Presentism in profile 3, with participants roughly evenly split in terms of showing weak agreement or disagreement to Presentism. Profile 1 is quite different from the other two. The majority of profile 1 participants agreed that the future was open, but not as strongly as participants in the other two profiles (compare top panels in Fig. 4). The majority of profile 1 participants also agreed that the past was not mutable, but again notably more weakly than the other two profiles (compare second row of panels in Fig. 4). The majority of profile 1 participants did not endorse Presentism, but there was quite wide variability in responding on this factor. Finally, unlike for profiles 2 and 3, there was a notable minority (37%) who did not agree that time has a direction, and even among profile 1 participants who did agree time had a direction, this was typically endorsed weakly. Statistical analyses of profile differences are reported in the Supplementary Materials.

In summary, profile 2 and 3 participants were similar in that they typically endorsed the future as open (with profile 2 endorsing to a greater extent than profile 3), the past as immutable, and time as having a direction, but differed in terms of endorsement of Presentism, which only profile 3 participants endorsed to any degree. profile 1 participants more weakly endorsed the future as open and the past as fixed, typically rejected Presentism although often weakly, and were distinctive in that they typically either only relatively weakly endorsed time as having a direction or denied the directionality of time.

Finally, in order to explore whether membership of specific profiles might be related to exposure to scientific discussion of the nature of time, we examined the relationship between profile membership and participants’ responses to the statement “Within the last three years I have read, watched, or listened to something about how scientists think time works.” Response options were “Never,” “Once,” “Two or three times,” and “More than two or three times.” A multinomial logistic regression was performed to explore the relation between profile and exposure to media information about how scientists think time works. The model was statistically significant (*χ*2(2) = 9.57, *p* = .008) and explained 4.2% (Nagelkerke *R* ^2^) of the variance. Increasing exposure to media information about how scientists think time works was associated with an increased likelihood of classification into profile 1 rather than profile 3 (Wald = 8.86, *β* = 0.368, Exp (*B*) = 1.44 [1.13, 1.84], *p* = .003), and approached, but did not reach, a significant association with classification into profile 1 rather than profile 2 (*p* = .064). There was no association between exposure to media information about how scientists think time works and membership of profile 2 versus profile 3 (*p* = .426).

An ordinal logistic regression analysis indicated that across profiles, factor scores for Directionality were significantly associated with exposure to scientific views about time, with an odds ratio of 1.38 [1.03, 1.85], Wald *χ*2(4) = 4.68, *p* = .030. Lower scores on the Directionality factor were associated with an increase in the odds of reporting exposure to scientific views about time in the media. Scores on the other three factors were not associated with the odds of reporting exposure to scientific views about time in the media (all *p*s > .173).

### 3.4 Discussion

In study 2, we tested the hypothesis that there is a relationship between the four latent belief constructs identified in study 1 (Open Future, Mutable Past, Presentism, and Directionality) and the individual beliefs that were said to reflect them. This four‐factor model of people's beliefs about time was a good fit for new data, providing good evidence for a relationship between the four proposed latent factors and the sets of individual beliefs from which they were extrapolated. We then used these four latent beliefs to examine the typology of people's beliefs about time, finding three profiles that suggest qualitatively different intuitive theories.

That no single intuitive theory of time emerged is significant, as this finding appears to go against the views of those philosophers who have suggested that people hold a particular set of beliefs that we can pick out collectively as “common sense time.” While the majority of participants (profiles 2 and 3; approximately 60%) responded in a way that was broadly consistent with three central features specified by philosophers to be components of common sense time, endorsing Open Future and Directionality and rejecting Mutable Past, these participants differed in terms of whether they endorsed (profile 3) or rejected (profile 2) the Presentist claim that some philosophers have considered to be a further feature of common sense time. Furthermore, a significant minority of participants (profile 1; approximately 40%) appeared to demonstrate an even greater divergence from “common sense time,” rejecting Presentism, but also displaying a significantly weaker endorsement of Open Future and Directionality and a greater endorsement of Mutable Past––to the extent that some participants believed that time did not have a direction and some also believed that the past is mutable.

## 4 General discussion

To the best of our knowledge, the current studies are the first to systematically examine the components of people's everyday beliefs about time. Although the studies were highly exploratory, the findings across the two studies were consistent and provide a useful starting point in attempts to characterize intutive theories of time.

In study 1, we presented participants with a large number of statements about time and explored their responses in relation to assumptions drawn from philosophical discussions: the Objective Now Assumption (the belief that, objectively, there is a moment in time that is the present moment, rather than which moment is “now” being merely a matter of one's perspective in time); the Past–Present–Future Difference Assumption (the belief that the past, the present, and the future are fundamentally different in nature); and the Dynamicity Assumption (the belief that time is something dynamic). In addition to examining whether participants agreed with specific statements, in our analysis, we used the technique of EFA in order to look for evidence of underlying dimensions in patterns of beliefs.

The factor analysis demonstrated that subsets of people's beliefs about time covary in interpretable ways, suggesting that they are associated with a smaller number of latent belief constructs, and that these constructs are involved in shaping people's intuitive theory of time. These four dimensions (Open Future, Mutable Past, Presentism, and Directionality) each reflect themes in philosophical discussions of time, as many philosophers take one or more, if not all, of these dimensions to be central to people's common sense view of time. The statements that loaded on Open Future addressed the knowability and settledness of the future, our ability to control the future and to do things now that influence it, as well as supposed differences in nature between the present and future; statements that loaded onto Mutable Past addressed control of and agency over the past; and statements that loaded onto Presentism addressed the reality of the future and past compared to the present (see Table 2 for the full list of statements). Each of these factors is thus related to the Past–Present–Future Difference Assumption highlighted previously. The statements that loaded onto Directionality addressed the question of whether there was a direction to time; this factor was thus related to the Past–Present–Future Difference and the Dynamicity Assumptions, explained above.

In study 2, we replicated this model using a new sample of adults, and found evidence of three mutually incompatible intuitive theories of time. Rather than a single intuitive theory emerging, we demonstrated the plausibility of three distinct profiles, with profiles 2 and 3 most closely aligning to philosophers’ characterizations of “common sense time.” Profile 2, but not profile 3, differed primarily from the way in which common sense time has been described insofar as participants with profile 2 showed a clear rejection of Presentism. Profile 1 participants, on the other hand, did not show a profile that was well‐matched to the claims made about common sense time, insofar as Open Future and Directionality were relatively weakly endorsed (the latter particularly so, and indeed in some instances rejected), Mutable Past was weakly rejected (and in some instances endorsed), and Presentism was rejected. While the majority of participants fell within profiles 2 and 3 (approximately 60%)––responding differently across the two profiles, though overall in a way that was broadly consistent with most aspects of common sense time––a significant minority of participants fell into profile 1 (approximately 40%; in fact, this was the most common individual profile). These differences between profiles are broadly consistent with Latham et al.’s (2021a) finding of a split between participants who did judge time to be dynamical and those who did not, discussed in Section 1.4.

Our results have a number of interesting implications. We noted at the outset that it is apparent that philosophers do not always agree about precisely which beliefs contribute to people's intuitive theory of time. This appears to be reflected in our evidence of multiple and, at least on the face of it, mutually incompatible intuitive theories of time held by three distinct subpopulations. As we now describe, we tentatively suggest that these subpopulations may (perhaps roughly) map onto different philosophical views of the nature of time.

### 4.1 Mapping profiles to philosophical views of time

The philosophical account of time called Presentism is often characterized as a view on which “presence is existence” (Tallant, 2014, p. 494), or “only present objects exist” (Markosian, 2004, p. 47; see also Bigelow, 1996; Bergmann, 1999; Crisp, 2004a, 2004b; Merricks, 2007). These claims appear to capture those beliefs expressed by participants in profile 3, insofar as the majority endorse the factor that we have called Presentism (relating to the existence of things in the past, present, and future), as well as endorsing Open Future and Directionality and rejecting Mutable Past.

Another philosophical account of time is called the Moving Spotlight view. According to C. D. Broad (1923, p. 59), people typically “regard the history of the world as existing eternally in a certain order of events. Along this, and in a fixed direction, we imagine the characteristic of presentness as moving, somewhat like the spot of light from a policeman's bull's‐eye traversing the fronts of the houses in a street. What is illuminated is the present, what has been illuminated is the past, and what has not yet been illuminated is the future…” (see also Craig [2000], p. 131–132). The picture presented by Broad is a view on which past, present, and future things are equally real (contrary to the Presentist view just discussed); yet, there is also a single moment of time that is objectively present, and that objective present moves along successive events in the past‐to‐future direction of time. While Broad does not endorse this view of time himself, he takes the Moving Spotlight view to be the one that people are “naturally tempted” toward (Broad, 1923, p. 59). Participants within profile 2 might be seen to hold a similar view, insofar as they reject Presentism and Mutable Past, but endorse Open Future and Directionality. Alternatively, participants within profile 2 might seem to be committed to a different “Eternalist” view—one in which the past, present, and future are all equally real, and time has a direction, but without there being any particular moment in time that is objectively present. Maudlin (2002, p. 259), for example, argues that time has an intrinsic direction, and this asymmetry accounts for the “passage” of time—without there being a moment that is objectively now. He takes such a view to be “part of common‐sense.” Given that none of the statements in study 1 that probed Objective Now beliefs featured in the EFA solution, and that responses to these statements tended to yield means close to 50 (Appendix B), discriminating between these two options would require further work on how best to frame questions to participants about the Objective Now.

Finally, participants in profile 1 appear to reject aspects of common sense time as it was characterized above, rejecting Presentism, and displaying a strikingly weaker endorsement of Open Future and Directionality, and weaker rejection of Mutable Past, when compared with profiles 2 and 3. We have suggested that this subpopulation might hold an intuitive theory of time that is more consistent with the scientific picture of time than the other two profiles (see, e.g., Mellor, 1998; Price, 1997), though here again, more work would be required to explore this and alternative possibilities more systematically.

### 4.2 Implications of the findings

Our findings are preliminary in that, as we acknowledge, this was a highly exploratory study. In future work, it will be important to seek evidence that our measure behaves as anticipated by, for instance, examining the relationship between profile membership and people's responses to vignettes that capture inferences about how time behaves in a world, or the relationship between profile membership and behaviors that reveal temporal biases (on the latter, see, e.g., Hoerl et al., 2022). Further, it will be important to consider whether the proportion of participants belonging to each profile, or the profiles themselves, differ across populations and cultures. Indeed, the issue of whether notions of time differ cross‐culturally is a long‐standing one in anthropology (Gell, 1992 [2020]). Due to the method of recruitment and the need for participants to be fluent in English, the vast majority of participants were most likely from Western, educated, industrialized, rich, and democratic societies (Henrich et al., 2010), and, therefore, it cannot be assumed that the findings would generalize across all cultures.

Nevertheless, the findings of study 1 and 2 were consistent in two important respects. First, for each statement that was used across both studies, a similar percentage of participants agreed and disagreed with the statements in studies 1 and 2, with most differences between 1% and 5% and the largest difference standing at 14%. Thus, the basic findings appear to be replicable. Second, the confirmatory factor analysis in study 2 confirmed the findings of the EFA in study 1, and the factors identified by these analyses were interpretable. What is the significance of our findings for philosophers and psychologists? As we outlined in the Introduction, some philosophers take people's intuitive theory of time to tell us something about how time really is. While different philosophical theories of time might be read as mapping on to the intuitive theories held by distinct subpopulations, our results suggest that in fact there is no one such intuitive theory of time with which people typically operate. Given such differences between subpopulations, our findings highlight the need for theorists to proceed with caution when invoking alleged intuitions about time.

Our findings are also significant for psychologists in several ways. Understanding more about any domain of intuitive belief provides an opportunity for exploring the ways in which intuitive theories interact with new and counterintuitive knowledge. New conceptual structures generated during discourse and education often compete with, rather than fully replacing, intuitive theories (Shtulman & Valcarcel, 2012; Vosniadou & Brewer, 1994), and mediate the ability to grasp the new theory (Fischbein et al., 1985). This is the case not only in childhood when scientific theories are first acquired (Vosniadou, 2013), but into adolescence and older adulthood (Kavanagh & Sneider, 2007; Pine et al., 2001; Stein et al., 2008), including among professional scientists (Shtulman & Harrington, 2016). The processes involved in revising intuitive beliefs about time may be particularly interesting because they have the potential to be unusual. For example, given an erroneous understanding of the trajectory of a falling object, it is possible to observe evidence to the contrary and thus revise the erroneous understanding (McCloskey et al., 1983). If people are wrong in fundamental ways about time, however, they are arguably not directly confronted with the inability of their theory to predict the world around them: there does not appear to be an aspect of people's everyday experience which, once attention has been drawn to it, could demonstrate to an individual that, for example, they were incorrect to believe that the past and future are different in nature to the present.

If people's beliefs about time really are theory‐like, and if we are correct in our delineation of multiple intuitive theories of time, there are further questions to be addressed concerning the role of exposure to scientific views of time in intuitive theory revision. Recall that participants in profile 1––who do not endorse some of what have been described as the core elements of common sense time––tended to have higher exposure scores than participants in profile 3 (when comparing participants in profile 1 with those in profile 2, this relationship approached, but did not reach, significance). Thus, a tentative possibility is that some profile 1 members hold theories about time that have been influenced by scientific views on time encountered in media (though not, on current evidence, by formal education; we found no association between level of education and profile membership). Alternatively, some profile 1 members may, prior to exposure to scientific views about time, be predisposed to an intuitive theory of time that is at least somewhat in alignment with those views. Were this the case, profile 1 members who have not had significant media exposure to scientific views about time might find it easier to acquire at least some components of a more formal conceptual grasp of “scientific time” than do people whose intuitive theory of time aligns more closely with so‐called common sense time (profiles 2 and 3), a possibility that could be tested using intervention studies.

What advantages might there be to understanding the discrepancies between the ways in which people naively think about time and our scientific understanding of time? One possibility is that this knowledge could potentially help devise strategies to help people to avoid temporal biases, such as valuing events more when they are located in the future rather than the past (Caruso et al., 2008; Caruso et al., 2013; Hoerl et al., 2022), and preferring unpleasant experiences to be located in the past and pleasant experiences in the future (Lee et al., 2020; Parfit, 1984; Sullivan, 2018). Some philosophers have argued that such time biases are irrational and could lead to decisions that ultimately hamper people's well‐being (e.g., Dougherty, 2015; Greene & Sullivan, 2015; Sullivan, 2018). If we accept these arguments, then it is important to establish a better picture of what people believe about the past and future, and just how this might determine the temporal biases they have (perhaps among other factors), so that we have a clearer idea of what it is that must be overcome.

Psychologists with an interest in individual differences in people's understanding of the world around them might also ask how individual differences in beliefs about time, as evidenced by the three profiles described by our data, can arise. Are there any aspects of people's lived experience, or of their wider systems of belief, that might explain the willingness of people belonging to profile 1 to reject some of the assumptions that have been thought to be characteristic of common sense time? And how might they be connected to other interesting individual differences, for example, in how people understand their own persistence and identity over time?––see, for instance, Velleman's (2006) suggestion that the sense people have of time passing is tied to the sense people have of how they themselves persist over time.

As important as the above considerations are, there is clearly also an independent and purely curiosity‐driven interest in how, precisely, people conceive of time. As Callender (2017, p. 1) says, “Time is a big invisible thing that will kill you. For that reason alone, one might be curious about what it is.” By the same token, one might be curious about how far, and in what ways, people's beliefs about time depart from science's best understanding of time––a phenomenon that pervades all aspects of people's lives and structures both their day‐to‐day interactions and decision making in such fundamental ways.

### Open Research Badges

This article has earned Open Data and Open Materials badges. Data and materials are available at https://osf.io/qng93/.

## Supporting information

Supporting Information

## Notes

1 Note that, within the context of this paper, use of the term “intuitive” does not presuppose that the relevant theories are instead innate or the product of early development. We take this to be a separate question.

2 While one heuristic for evaluating factor solutions holds that the eigenvalue for each factor must be equal to or greater than 1 (Kaiser, 1960), this may exclude theoretically important factors, since factor analysis explains only common variance, unique variance may be unevenly distributed across variables, and this may vary across samples (Horn, 1965).

## Appendix A Proportion of participants who demonstrated agreement with each statement (score of 51 or above) for Beliefs About Time statements, study 1. Statements are presented in pairs (i.e., as a statement and its converse in adjacent and identically shaded lines) and grouped according to the most relevant assumption to which they relate.

**Table:**

Statement	*n*	*%* agreement (score *≥* 51)
Objective Now Assumption		
When an event turns from being future to being present to being past, nothing about the event itself changes	211	48.82%
When an event turns from being future to being present to being past, something about the event itself changes as well	207	67.63%
Whether an event is past, present, or future is just our perspective on it rather than a fact about that event itself	215	55.35%
Whether an event is past, present, or future is a fact about that event itself rather than just our perspective on it	211	61.14%
Past–Present–Future Difference Assumption		
Subcategory: Reality and Fundamental Nature		
Things in the future are real	206	67.48%
Things in the future are not real	213	40.38%
Things in the past are real, even if they are not in the present	218	90.83%
Things in the past are not real, unless they are also in the present	219	16.89%
Things in the past are real, but not in the same way as things in the present	217	66.82%
Things in the past are real in the same way as things in the present	219	73.52%
Things in the future are real, but not in the same way as things in the present	213	73.24%
Things in the future are real in the same way as things in the present	216	46.76%
Things are only real if they are in the present	220	27.27%
Things in the past and the future are just as real as things in the present	219	63.47%
The past and present are not fundamentally different in nature	215	49.30%
The past and present are fundamentally different in nature	215	74.42%
The present and future are not fundamentally different in nature	213	43.66%
The present and future are fundamentally different in nature	215	76.74%
Subcategory: Control and Certainty		
We have some control now over what will happen in the future	214	94.39%
We have no control now over what will happen in the future	218	24.31%
We have some control now over what has happened in the past	219	30.59%
We have no control now over what has happened in the past	221	82.81%
Things can be done now that change what will happen in the future	217	97.70%
Nothing can be done now that changes what will happen in the future	218	13.76%
Things can be done now that change what has happened in the past	219	24.20%
Nothing can be done now that changes what has happened in the past	219	79.45%
Not only can we change how we think or feel about the past, but we can also change what actually happened	218	17.43%
We can change how we think or feel about the past but not what actually happened	219	90.87%
Which way the future will go is determined by what has already happened	216	71.76%
Which way the future will go is not determined by what has already happened	218	36.24%
The future is already settled	215	14.42%
The future is not yet settled	215	90.23%
We can always have more knowledge about the past than we can about the future	218	90.83%
It is possible to have as much knowledge about the future as we can have about the past	219	15.98%
The past and future are equally certain	217	19.35%
The past and future are not equally certain	214	72.90%
Dynamicity Assumption		
Subcategory: Directionality		
Time flows forward, but it could also flow backward	208	31.25%
Time only flows forward. It could never flow backward	212	78.77%
Time has a direction	205	82.44%
Time does not have a direction	211	30.81%
Time flows forward	209	89.47%
Time does not flow forward	215	14.88%
It is not possible for time to reverse its direction	203	76.35%
It could be possible for time to reverse its direction	206	25.73%
Subcategory: Time Passing/Flowing		
Time is moving in relation to us	203	64.04%
Time is not moving in relation to us	199	51.76%
We are moving in relation to time	207	76.33%
We are not moving in relation to time	192	31.77%
Things pass from future to present to past	211	55.92%
Things do not pass from future to present to past	208	41.35%
The present moves forward in time	212	75.00%
The present does not move forward in time	208	42.79%
Things in the future move toward us	209	63.16%
Things in the future do not move toward us	204	44.61%
Things in the past move away from us	217	74.19%
Things in the past do not move away from us	212	43.87%
The statement “time flows” really means “Things move from being in the future to being in the present to being in the past”	207	66.18%
The statement “time flows” really means something other than “Things move from being in the future to being in the present to being in the past”	202	51.49%
The statement “time flows” really means “What time is 'now’ changes”	205	72.68%
The statement “time flows” really means something other than “What time is 'now’ changes”	200	51.50%
The statement “time flows” really means “Different things happen at different times”	208	59.62%
The statement “time flows” really means something other than “Different things happen at different times”	201	66.67%
The statement “time flows” really means “One thing happens at one time, another thing happens at another time”	209	63.64%
The statement “time flows” really means something other than “One thing happens at one time, another thing happens at another time”	202	58.42%
The statement “time flows” really means “What is real changes”	206	50%
The statement “time flows” really means something other than “What is real changes”	204	61.76%

*Note*. While one statement of each pair was reverse‐scored for the purposes of analyses, original response values are presented here.

## Appendix B Descriptive statistics for Beliefs About Time statements, study 1

**Table:**

Statement	*n*	*M (SD)*	95% CI	Min	Max	Spearman–Brown split‐half reliability
Objective Now Assumption						
When an event turns from being future to being present to being past, nothing about the event itself changes	214	50.36 (30.99)	46.18, 54.54	0	100	0.34
When an event turns from being future to being present to being past, something about the event itself changes as well	213	57.62 (27.81)	53.87, 61.38	0	100	
Whether an event is past, present, or future is just our perspective on it rather than a fact about that event itself	216	50.91 (32.18)	46.60, 55.23	0	100	0.21
Whether an event is past, present, or future is a fact about that event itself rather than just our perspective on it	214	54.32 (31.37)	50.08, 58.56	0	100	
Past–Present–Future Difference Assumption						
Subcategory: Reality and Fundamental Nature						
Things in the future are real	212	60.26 (29.51)	56.26, 64.26	0	100	0.64
Things in the future are not real	214	41.85 (31.41)	37.61, 46.08	0	100	
Things in the past are real, even if they are not in the present	219	81.64 (22.11)	78.69, 84.58	0	100	0.66
Things in the past are not real, unless they are also in the present	220	22.61 (27.06)	19.02, 26.21	0	100	
Things in the past are real, but not in the same way as things in the present	218	57.8 (30.51)	53.73, 61.87	0	100	0.37
Things in the past are real in the same way as things in the present	220	68.34 (29.05)	64.48, 72.20	0	100	
Things in the future are real, but not in the same way as things in the present	216	62.32 (28.78)	58.46, 66.18	0	100	−0.45
Things in the future are real in the same way as things in the present	217	48.40 (31.21)	44.22, 52.57	0	100	
Things are only real if they are in the present	221	32.55 (30.80)	28.47, 36.64	0	100	0.27
Things in the past and the future are just as real as things in the present	221	60.49 (31.63)	56.30, 64.68	0	100	
The past and present are not fundamentally different in nature	217	46.09 (29.59)	42.13, 50.05	0	100	0.47
The past and present are fundamentally different in nature	216	63.18 (27.40)	59.51, 66.86	0	100	
The present and future are not fundamentally different in nature	214	45.62 (28.96)	41.71, 49.52	0	100	0.36
The present and future are fundamentally different in nature	217	67.62 (28.02)	63.87, 71.37	0	100	
Subcategory: Control and Certainty						
We have some control now over what will happen in the future	217	78.93 (19.56)	76.31, 81.54	0	100	0.58
We have no control now over what will happen in the future	220	32.75 (29.67)	28.81, 36.70	0	100	
We have some control now over what has happened in the past	221	28.95 (30.32)	24.92, 32.98	0	100	0.65
We have no control now over what has happened in the past	221	76.64 (26.98)	73.07, 80.22	0	100	
Things can be done now that change what will happen in the future	219	85.73 (16.05)	83.59, 87.87	10	100	0.52
Nothing can be done now that changes what will happen in the future	220	22.54 (26.44)	19.02, 26.06	0	100	
Things can be done now that change what has happened in the past	220	26.01 (30.28)	21.99, 30.03	0	100	0.56
Nothing can be done now that changes what has happened in the past	220	76.22 (28.33)	72.46, 79.99	0	100	
Not only can we change how we think or feel about the past, but we can also change what actually happened	218	20.86 (27.38)	17.20, 24.51	0	100	0.58
We can change how we think or feel about the past but not what actually happened	220	85.34 (22.15)	82.40, 88.28	0	100	
Which way the future will go is determined by what has already happened	218	59.92 (27.34)	56.27, 63.57	0	100	0.36
Which way the future will go is not determined by what has already happened	219	39.85 (30.44)	35.79, 43.92	0	100	
The future is already settled	217	21.76 (25.68)	18.32, 25.19	0	100	0.72
The future is not yet settled	217	81.15 (23.87)	77.96, 84.35	0	100	
We can always have more knowledge about the past than we can about the future	219	81.97 (22.00)	79.04, 84.90	0	100	0.51
It is possible to have as much knowledge about the future as we can have about the past	220	24.06 (24.97)	20.75, 27.38	0	100	
The past and future are equally certain	217	27.68 (30.33)	23.62, 31.74	0	100	0.35
The past and future are not equally certain	216	67.17 (32.36)	62.83, 71.51	0	100	
Dynamicity Assumption						
Subcategory: Directionality						
Time flows forward, but it could also flow backward	211	33.87 (31.71)	29.56, 38.17	0	100	0.76
Time only flows forward. It could never flow backward	213	71.30 (29.62)	67.29, 75.30	0	100	
Time has a direction	206	71.55 (29.23)	67.53, 75.56	0	100	0.58
Time does not have a direction	212	35.29 (33.53)	30.75, 39.83	0	100	
Time flows forward	212	76.97 (23.34)	73.81, 80.13	0	100	0.45
Time does not flow forward	216	24.89 (26.42)	21.35, 28.44	0	100	
It is not possible for time to reverse its direction	203	71.72 (30.83)	67.46, 75.99	0	100	0.67
It could be possible for time to reverse its direction	208	28.45 (30.55)	24.27, 32.62	0	100	
Subcategory: Time Passing/Flowing						
Time is moving in relation to us	204	54.54 (31.5)	50.19, 58.89	0	100	0.43
Time is not moving in relation to us	205	51.03 (34.51)	46.27, 55.80	0	100	
We are moving in relation to time	212	64.26 (26.39)	60.69, 67.84	0	100	0.59
We are not moving in relation to time	197	38.31 (28.15)	34.36, 42.27	0	100	
Things pass from future to present to past	213	50.99 (36.15)	46.11, 55.87	0	100	0.57
Things do not pass from future to present to past	212	44.67 (35.95)	39.80, 49.54	0	100	
The present moves forward in time	213	66.53 (29.06)	62.60, 70.45	0	100	0.60
The present does not move forward in time	208	43.13 (32.21)	38.73, 47.53	0	100	
Things in the future move toward us	211	55.35 (30.39)	51.22, 59.47	0	100	0.58
Things in the future do not move toward us	211	47.80 (29.21)	43.83, 51.76	0	100	
Things in the past move away from us	218	64.59 (27.38)	60.94, 68.25	0	100	0.43
Things in the past do not move away from us	214	48.34 (29.87)	44.31, 52.36	0	100	
The statement “time flows” really means “Things move from being in the future to being in the present tobeing in the past”	209	58.45 (31.24)	54.19, 62.71	0	100	−0.13
The statement “time flows” really means something other than “Things move from being in the future to being in the present to being in the past”	204	50.64 (29.55)	46.56, 54.72	0	100	
The statement “time flows” really means ‘What time is “now’ changes”	210	61.59 (26.11)	58.03, 65.14	0	100	−0.03
The statement “time flows” really means something other than “What time is 'now’ changes”	202	50.04 (27.13)	46.28, 53.80	0	100	
The statement “time flows” really means “Different things happen at different times”	208	52.30 (27.90)	48.49, 56.12	0	100	0.17
The statement “time flows” really means something other than “Different things happen at different times”	203	58.78 (28.59)	54.83, 62.74	0	100	
The statement “time flows” really means “One thing happens at one time, another thing happens at another time”	210	55.98 (28.58)	52.09, 59.87	0	100	−0.14
The statement “time flows” really means something other than “One thing happens at one time, another thing happens at another time”	204	54.38 (28.96)	50.38, 58.38	0	100	
The statement “time flows” really means “What is real changes”	207	48.27 (28.70)	44.33, 52.20	0	100	−0.15
The statement “time flows” really means something other than “What is real changes”	205	57.66 (27.27)	53.90, 61.41	0	100	

*Note*. While one statement of each pair was reverse‐scored for the purposes of analyses, original means, minimum and maximum values, and 95% confidence intervals are presented here. Split‐half coefficients, however, are presented for pairs of variables following reverse‐scoring of one of each variable pair.

## Appendix C Descriptive statistics for Beliefs About Time statements, study 2

**Table:**

Factor	Statement	*n*	*M (SD)*	Min	Max	95% CI	Spearman–Brown split‐half reliability
Open Future	F1: We can always have more knowledge about the past than we can about the future	249	80.86 (20.19)	0	100	78.34, 83.38	N/A
Open Future	F2: The future is not yet settled	249	84.54 (20.85)	0	100	81.94, 87.14	N/A
Open Future	F3: Things can be done now that change what will happen in the future	250	87.55 (18.04)	0	100	85.30, 89.80	0.813
Open Future	F4: Nothing can be done now that changes what will happen in the future	250	13.89 (19.32)	0	100	11.48, 16.29	0.813
Open Future	F5: We have no control now over what will happen in the future	250	28.52 (28.88)	0	100	24.92, 32.12	N/A
Open Future	F6: The present and future are fundamentally different in nature	231	65.48 (28.29)	0	100	61.81, 69.15	N/A
Mutable Past	M1: Not only can we change how we think or feel about the past, but we can also change what actually happened	248	16.69 (25.37)	0	100	13.52, 19.87	N/A
Mutable Past	M2: Things can be done now that change what has happened in the past	249	22.34 (29.12)	0	100	18.71, 25.98	N/A
Mutable Past	M3: We have some control now over what has happened in the past	249	23.86 (27.20)	0	100	20.46, 27.25	0.414
Mutable Past	M4: We have no control now over what has happened in the past	250	78.24 (28.51)	0	100	74.69, 81.79	0.414
Presentism	P1: Things in the future are real	244	65.48 (27.59)	0	100	62.00, 68.96	0.804
Presentism	P2: Things in the future are not real	242	32.22 (30.40)	0	100	28.37, 36.07	0.804
Presentism	P3: Things in the past and the future are just as real as things in the present	249	64.10 (29.91)	0	100	60.37, 67.84	N/A
Presentism	P4: Things in the future are real in the same way as things in the present	246	53.89 (31.56)	0	100	49.93, 57.86	N/A
Directionality	D1: It is not possible for time to reverse its direction	223	76.56 (29.75)	0	100	72.63, 80.48	N/A
Directionality	D2: It could be possible for time to reverse its direction	226	24.55 (27.99)	0	100	20.88, 28.22	N/A
Directionality	D3: Time has a direction	226	76.70 (25.01)	0	100	73.42, 79.98	N/A
Directionality	D4: Time only flows forward. It could never flow backward	230	81.56 (25.40)	0	100	78.26, 84.86	N/A

*Note*. While one statement of each pair was reverse‐scored for the purposes of analyses, original means, minimum and maximum values, and 95% confidence intervals are presented here. Split‐half coefficients, however, are presented for pairs of variables following reverse‐scoring of one of each variable pair.

## Appendix D Proportion of participants who demonstrated agreement with each statement (>51), study 2

**Table:**

Factor	Statement	*n*	*%* agreement (score *≥* 51)
Open Future	F1: We can always have more knowledge about the past than we can about the future	241	93.36%
Open Future	F2: The future is not yet settled	249	93.98%
Open Future	F3: Things can be done now that change what will happen in the future	249	95.58%
Open Future	F4: Nothing can be done now that changes what will happen in the future	247	6.48%
Open Future	F5: We have no control now over what will happen in the future	247	20.65%
Open Future	F6: The present and future are fundamentally different in nature	226	73.00%
Mutable Past	M1: Not only can we change how we think or feel about the past, but we can also change what actually happened	247	12.55%
Mutable Past	M2: Things can be done now that change what has happened in the past	248	21.77%
Mutable Past	M3: We have some control now over what has happened in the past	245	22.86%
Mutable Past	M4: We have no control now over what has happened in the past	246	83.74%
Presentism	P1: Things in the future are real	239	74.48%
Presentism	P2: Things in the future are not real	242	26.45%
Presentism	P3: Things in the past and the future are just as real as things in the present	244	70.08%
Presentism	P4: Things in the future are real in the same way as things in the present	243	55.14%
Directionality	D1: It is not possible for time to reverse its direction	217	80.65%
Directionality	D2: It could be possible for time to reverse its direction	214	21.96%
Directionality	D3: Time has a direction	216	90.28%
Directionality	D4: Time only flows forward. It could never flow backward	222	90.09%

*Note*. While one statement of each pair was reverse‐scored for the purposes of analyses, original response values are presented here.

## Appendix E Unstandardized estimates, standard errors, Wald statistic, and significance values for each indicator, study 2

**Table:**

Factor		β	SE	Z	*p*
Open Future	F1: We can always have more knowledge about the past than we can about the future	5.51​	1.91​	2.88​	.004**​
Open Future	F2: The future is not yet settled	3.21​	1.58​	2.03​	.042*​
Open Future	F3: Things can be done now that change what will happen in the future	16.42​	1.90​	8.63​	< .001***​
Open Future	F4: Nothing can be done now that changes what will happen in the future	14.60​	1.77​	8.24​	< .001***​
Open Future	F5: We have no control now over what will happen in the future	10.02​	1.73​	5.79​	< .001***​
Open Future	F6: The present and future are fundamentally different in nature	2.40​	2.10​	1.14​	.253​
Mutable Past	M1: Not only can we change how we think or feel about the past, but we can also change what actually happened	13.27​	2.04​	6.49​	< .001***​
Mutable Past	M2: Things can be done now that change what has happened in the past	19.12​	2.15​	8.91​	< .001***​
Mutable Past	M3: We have some control now over what has happened in the past	16.15​	1.92​	8.41​	< .001***​
Mutable Past	M4: We have no control now over what has happened in the past	11.65​	2.06​	5.65​	< .001***​
Presentism	P1: Things in the future are real	23.65​​	1.51​​	15.71​​	< .001***​​
Presentism	P2: Things in the future are not real	23.15​​	1.58​​	14.62​​	< .001***​​
Presentism	P3: Things in the past and the future are just as real as things in the present	18.40​​	1.78​​	10.35​​	< .001***​​
Presentism	P4: Things in the future are real in the same way as things in the present	24.21​​	1.55​​	15.61​​	< .001***​​
Directionality	D1: It is not possible for time to reverse its direction	27.42​	1.49​	18.36​	< .001*** ​
Directionality	D2: It could be possible for time to reverse its direction	18.40​	1.74​	10.57​	< .001*** ​
Directionality	D3: Time has a direction	9.03​	1.94​	4.66​	< .001*** ​
Directionality	D4: Time only flows forward. It could never flow backward	18.89	1.91​	9.92​	< .001*** ​

* *p* < .05; ** *p* < .01; *** *p* < .001.

## Appendix F Bivariate correlations between each of the indicator variables, study 2

**Table:**

	F1	F2	F3	F4	F5	F6	O1	O2	O3	O4	P1	P2	P3	P4	D1	D2	D3
F1	–																
F2	.10	–															
F3	.26***	.11	–														
F4	.18***	.16*	.69***	–													
F5	−.02	.14*	.30***	.34***	–												
F6	.17**	.11	.09	.01**	.05	–											
M1	−.01	−.11	−.17**	−.13*	−.12	−.12	–										
M2	−.13*	−.03	−.26***	−.19**	−.03	−.09	.37***	–									
M3	−.12	.01	−.31***	−.22***	−.14*	−.01	.29***	.37***	–								
M4	−.07	−.03	−.07	−.12	−.07	.09	.18**	.29***	.27***	–							
P1	.03	−.004	−.19**	−.08	−.09	.16*	−.01	−.13*	−.01	−.11	−						
P2	.05	.10	−.19**	−.04	−.04	.13*	.04	−.03	.10	−.05	.67***	−					
P3	.05	.01	−.01	.03	−.02	.08	−.01	−.08	−.08	−.01	.54***	.41***	−				
P4	.05	.07	−.11	.02	.01	.18**	−.11	−.15*	.01	−.07	.64***	.60***	.52***	−			
D1	.14*	.13*	.19**	.14*	.07	.05	−.30***	−.23***	−.24***	−.21**	.09	.12	.08	.24***	−		
D2	.31***	.04	.22***	.13*	.07	.06	−.19**	−.24***	−.23***	−.11	−.05	−.01	.06	.04	.58***	−	
D3	.08	.07	.06	.05	.04	.09	−.20**	−.07	−.15*	−.05	.10	.06	.02	.06	.35***	.21**	−
D4	.19**	.06	.12	.15*	−.05	.19**	−.16*	−.21**	−.18**	−.08	.06	.10	.04	.19**	.66***	.52***	.22**

* *p* < .05; ** *p* < .01; *** *p* < .001.
