# Supplementary material for: Toward an Account of Intuitive Time
Source: Cogn Sci. 2022 Jun 22;46(7):e13166. doi: 10.1111/cogs.13166 (PMC9286814; doi:10.1111/cogs.13166)
Supplement: Supplementary file 1 — Supporting Information [file COGS-46-0-s001.docx]

**Towards an account of intuitive time**

**Supplementary materials**

**Participants, Studies 1 and 2**

**Study 1**

The sample was multinational. Approximately half of participants (48.86%) reported a level of education below a Bachelor’s degree or equivalent, 28.1% of participants reported holding a Bachelor’s degree or equivalent, 14.9% reported holding a Master’s degree or equivalent, and 4.5% reported holding a PhD or equivalent; 3.6% stated that they would rather not say.

**Study 2**

The sample was again multinational. Approximately 40% of participants (42.3%) reported a level of education below a Bachelor’s degree or equivalent, 35.5% of participants reported holding a Bachelor’s degree or equivalent, 15.5% reported holding a Master’s degree or equivalent, and 2.4% reported holding a PhD or equivalent; 4.4% stated that they would rather not say.

Education was not significantly associated with profile membership: *χ2* = 1.10, *df* = 1, *p* = .294.

**Generation of question sequences, Study 1**

Question sequences were generated using the Random Sequence Generator function provided at random.org (Haahr, 2019). When a question appeared in the random sequence within two questions of its converse, a further random sequence was generated, the question was located in this second sequence, and it was moved to this new place (e.g., 9^th^) in the original sequence. This procedure was repeated until no question appeared within two questions of its converse.

**Practice statement, Study 1**

Participants also responded to an additional initial practice statement, ‘Ripe bananas are delicious’, which was always presented first. When participants moved the slider in response to the practice statement, they received feedback based on the number they chose (0-25, ‘This means you dislike ripe bananas’; 25-49, ‘This means you don’t like ripe bananas all that much’; 50-74, ‘This means you quite like ripe bananas’; 75-100, ‘This means you really like ripe bananas’).

**Removal of pairs statements with low reliability, Study 1**

Pairs of statements (i.e., a statement plus its converse) demonstrated variable reliability (split-half coefficients between −.454 and .764). Five pairs of statements showed negative reliability. The first of these pairs comprised the statements ‘Things in the future are real in the same way as things in the present’ (positively worded) and ‘Things in the future are real, but not in the same way as things in the present’ (negatively worded). The latter, negatively phrased statement was excluded from the analysis. Upon examination of the remaining four pairs of statements demonstrating negative reliability, it was evident that all dealt with the flow of time. Each pair was phrased in a similar manner, comprising a statement about what ‘time flows’ really means, and a converse statement stating that ‘time flows’ really means something other than this (e.g., ‘The statement ‘time flows’ really means ‘Things move from being in the future to being in the present to being in the past’; ‘The statement ‘time flows’ really means something other than ‘Things move from being in the future to being in the present to being in the past’). Of these pairs, each negatively phrased statement was excluded from the analysis. One further pair of statements dealing with the flow of time was phrased in this manner, and demonstrated positive but low reliability. For consistency, the negatively phrased statement from this pair was also excluded from the analysis.

**Data imputation**

Factor scores are values for each participant representing where that participant lies on a scale representing the factor. Without complete data for a participant, it is not possible to obtain a factor score for that individual. Therefore, following descriptive analyses and before running factor analyses, we used the package “mice” (van Buuren & Groothuis-Oudshoorn, 2010) in R (R Core Team, 2018) to obtain a single set of values with which to substitute missing data resulting from Don’t Know responses. Most variables had skewed distributions, and Predictive Mean Matching (PMM) was therefore selected as the imputation method. PMM retains the original distribution of the data and makes no distributional assumptions, thus giving plausible interpretations for non-normally distributed data (van Buuren, 2018). We used a single imputation because while multiple imputation improves confidence intervals for population estimates, it cannot be straightforwardly implemented in the case of factor scores, since the signs of factors can arbitrarily ‘flip’ between positive and negative in different imputations.

To guard against the possibility of important discrepancies between the final 4-structure model and a four-factor model of the data with missing values (that is, the data without the imputed values that replaced ‘don’t know’ responses), the latter model was computed using the variable reduction procedure described above, and the two models were compared. The factor structure (which variables loaded onto which factors) was replicated exactly.

**Model selection, Study 1**

Exploratory factor analysis risks overestimating trivial factors (Gorsuch, 1983), and so requires both formal factor estimation criteria and consideration of the interpretability and conceptual clarity of factor structures. We first used the functions VSS and fa.parallel from the R package “psych” (Revelle, 2019), which produce suggestions for the optimal number of factors to retain. Each model can then be developed separately and evaluated on further formal and conceptual criteria. These criteria produced a number of factor estimates ranging from two (Very Simple Structure complexity) through four to six (scree-plot inspection), five (Velicer’s MAP criterion), to ten (parallel analysis, although a visual inspection of the parallel analysis scree plot suggested four factors).

Factor solutions for Beliefs About Time statements were computed for two, three, four, five, six, and ten factors, using the function “fa” from the R package “psych” (Revelle, 2019). This involved several steps for each solution. We first examined factor loadings, which are correlations between the variables that constitute a latent factor and the factor itself. Beliefs About Time statements that did not load onto any factor over the conventional value of .4 (Stevens, 1992) were removed from the analysis. During this process the 2- and 10-factor solutions encountered ultra-Heywood cases, which indicate that the sum of squared factor loadings for a variable, known as communalities, exceed 1; this indicates an inappropriate model. The two- and ten-factor models were rejected. Next, we removed variables that shared very little variance with the common factors, as measured by their communalities, which indicate the proportion of their variance that is contributed by the factors. Variables with communalities of less than .2 (Child, 2006) were considered of limited utility in identifying a common factor structure, and were removed incrementally. The remaining three-, four-, five-, and six-factor models each demonstrated strong factor loadings and no cross-loading variables.

We evaluated the three-, four-, five-, and six-factor models for interpretability. A minimum of three variables should load onto each factor for that factor to represent a stable latent construct (Tabachnick & Fidell, 2013). We assessed conservatively the number of distinct variables loading onto each factor by pairing variables that were the converse of one another. The five- and six-factor solutions each contained a factor onto which only two variables loaded; one of these variables was the converse of the other. These solutions were not considered further. Three or more distinct item pairs loaded onto each factor of the three- and four-factor solutions. We next compared the three- and four-factor solutions for interpretability. The four-factor solution contained interpretable distinctions that were not present in the three-factor solution, and the four-factor solution was accepted.

**Internal consistency and discriminant validity of the four-factor model, Study 1**

Five items in the initial four-factor solution had relatively low communalities of below .3, suggesting that they may not correlate well with the other items loading onto their respective factors. This may influence the reliability of the factors as subscales, as well as the ability of items to discriminate well between individuals at the extreme ends of the scale. Cronbach’s alpha was calculated for each factor using the function score.items in the R package “psych” (Revelle, 2019). Open Future achieved an alpha of .81, Mutable Past an alpha of .74, Presentism an alpha of .59, and Directionality an alpha of .74. We examined output from score.items reporting the reliability of each subscale if an item is dropped. Dropping the item with a communality below .3 from the Mutable Past subscale improved alpha from .74 to .81. Dropping two items with a communality below .3 from the Presentism subscale improved alpha from .59 to .72. No other variables had more than a negligible effect on the alpha for their subscale, including the two remaining items with communalities below .3. Since both of these items were a conceptual fit for their respective factors, they were retained. As a check on the advisability of dropping the three items that significantly impacted alpha, we also examined the correlation of the score for each item with the total score (excluding the item itself). Each of the dropped items (and none of the other items) had an item-subscale correlation below .3, suggesting that the item correlated poorly with the subscale overall (Field et al., 2012). The final four-factor solution therefore excluded these three items, and Cronbach’s alpha now exceeded .7 for each factor (Table S1).

A model with acceptable discriminant validity should demonstrate inter-factor correlations lower than the average inter-item correlations, and this was the case (Table S1).

Table S1

*Inter-factor correlations (below diagonal) and average inter-item correlations within each factor (diagonal)*

|  | Factor | 1 | 2 | 3 | 4 |
| --- | --- | --- | --- | --- | --- |
|  |  |  |  |  |  |
| 1 | Open Future | .41 |  |  |  |
| 2 | Mutable Past | -.43*** | .51 |  |  |
| 3 | Presentism | .11 | -.21** | .40 |  |
| 4 | Directionality | .08 | -.08 | .16** | .42 |

** p* < .05; *** p* < .01; **** p* < .001

**Model selection, confirmatory factor analysis, Study 2**

Several fit indices were calculated to assess the extent to which the data represented a fit for the specified four-factor model (see Brown, 2006). Goodness of fit was assessed using the Satorra-Bentler corrected χ2​ test, the χ2​/df ratio, robust Root Mean Square Error of Approximation (RMSEA), robust Comparative Fit Index (CFI), robust Tucker-Lewis Index (TLI) and the robust Standardized Root Mean Square Residual (SRMR). Table 6 shows the robust goodness-of-fit statistics for the four-factor model.

We first assessed absolute fit using the model chi-square (χ2​), which assesses the level of discrepancy between the sample and the fitted covariance matrix, and a relative χ2 statistic that divides the chi-square value by the degrees of freedom. This relative measure is influenced by sample size to a lesser degree than the model χ2​. Although the model χ2 test was significant, indicating a discrepancy between the sample and fitted covariance matrices (Brown, 2006), the χ2/df ratio was <2, suggesting that the model was a good fit for the data (Byrne, 1991). Furthermore, the SRMR, which reflects the standardized difference between observed and predicted correlation matrices, was < .07, indicating that the specified model was a good fit for the data (Hu & Bentler, 1999).

We next calculated comparative fit indices, which compare the model fit of specified models versus more restricted baseline models. Both the Comparative Fit Index (CFI: Bentler, 1990) and the more conservative Tucker-Lewis index, which penalizes for analyses involving more complex models (TLI: Tucker & Lewis, 1973) had values of >.90, indicating that the specified model fitted the data better than a more restricted baseline model (Hu & Bentler, 1999).

Finally, we calculated parsimony correction indices, which take into account any penalties accrued for poor model parsimony. The root mean square error of approximation (RMSEA; Steiger & Lind, 1980) reflects the discrepancy between the hypothesized model and the population covariance matrix (Hooper et al., 2008). For our model the RMSEA was .05, indicating good model fit (MacCallum et al., 1996).

**Proportion of participants endorsing each factor by profile, Study 2**

As a further way of inspecting the data, dichotomized raw factor scores were calculated for each factor by profile by categorizing scores at or above 51 as endorsement of the construct represented by the factor, and scores below 50 as non-endorsement. Scores at or above 50 and below 51 were excluded. The proportion of participants who endorsed each factor is reported by profile in Table S2.

Table S2

*Proportion of participants who endorsed each factor (score of 51 or above) by profile, Study 2, based on raw unweighted factor scores.*

| Factor | Profile 1 (*n* = 92) | Profile 2 (*n =* 59) | Profile 3 (*n =* 89) |
| --- | --- | --- | --- |
| Open Future | 92.31 | 100 | 98.88 |
| Mutable Past | 18.68 | 0 | 0 |
| Presentism | 25.27 | 0 | 56.18 |
| Directionality | 62.64 | 100 | 100 |

**Examination of cross-profile differences, Study 2**

In order to statistically examine cross-profile differences, a series of Bonferroni-corrected independent t-tests (*p* value set at 0.00416) were carried out to investigate the distinctiveness of the three profiles on each of the four latent factors. All of the profiles differed significantly from one another on Open Future and Presentism. On Open Future, Profile 3 (Mean = .20) scored significantly higher than Profile 1 (Mean = -.52) (*t* (129.81) = -5.56, *p* < .001). Profile 2 (Mean = .59) scored significantly higher than Profiles 1 (*t* (109.62) = -9.05, *p* < .001) and 3, (*t* (135.20) = 7.31, *p* < .001). For Presentism, Profile 3 (Mean = .59) scored significantly higher than Profile 1 (Mean = -.08) (*t* (190) = -5.36, *p* < .001) and Profile 2 (Mean = -.77), (*t* (132.09) = -13.51, *p* < .001). Profile 1 scored significantly higher than Profile 2 (*t* (146.15) = 7.00, *p* < .001). For Mutable Past, Profile 1 (Mean = .66) scored significantly higher than Profiles 2 (Mean = -.47), (*t* (153.92) = 10.88, *p* < .001) and 3 (Mean = -.43), (*t* (153.92) = 10.88, *p* < .001). Profile 3 scored higher than Profile 2, but this difference was not statistically significant (*t* (148) = -.45, *p* = .657). For Directionality, Profile 2 (Mean = .60) scored significantly higher than Profile 1 (Mean = -.93), (*t* (146.38) = -17.41, *p* < .001). Profile 3 (Mean = .64) also scored significantly higher than Profile 1 (*t* (124.73) = -19.24, *p* < .001). Finally, Profile 3 scored higher than Profile 2, but this difference was not statistically significant (*t* (105.13) = -.86, *p* = .368).

**Model specification and selection, latent profile analysis, Study 2**

**Model specification**

We specified the latent profile models to find profile solutions using four different methods of parametrization, as outlined by Masyn (2013); (1) a model in which the variances are equal and the covariances are set to zero (Model 1); (2) a model in which the variances are allowed to vary and the covariances are set to zero (Model 2); (3) a model in which both variances and covariances are equal (Model 3); (4) a model in which both variances and covariances are allowed to vary (Model 6). An analytic hierarchy process, based on the fit indices AIC, AWE, BIC, CLC, and KIC (see Akogul & Erisoglu, 2017) was generated by the tidyLPA package, which was used to determine the best profile solution for the data.

**Model selection**

Following Spurk and colleagues (Spurk et al., 2020), we first evaluated whether there was any evidence of errors or out of bound parameters. This was found to be the case when the number of specified profiles exceeded five. We therefore searched for solutions using all four methods of parametrization while capping the maximum number of profiles at five. Fit indices for each of the models under consideration are presented in Table S3. The hierarchical process indicated that the best solution was a five-profile solution generated using the parametrization of Model 2. However, this solution was not theoretically interpretable (Spurk et al., 2020), and one of the profiles contained less than 10% of the sample. The analysis was therefore re-run with the maximum number of solutions capped at four. The hierarchical process indicated that the best solution was a four-profile solution generated using the parametrization of Model 2. Again, however, this solution was not readily interpretable and contained a profile comprising less than 10% of the sample. The analysis was re-run, looking for solutions with a maximum of three profiles. This analysis yielded an interpretable three-profile solution, calculated using the parametrization of Model 2, with each profile containing more than 10% of the overall sample. This solution was accepted and contained 101 participants (40.24%) in Profile 1, 59 (23.5%) in Profile 2, and 91 (36.25%) in Profile 3.

Table S3

*Fit statistics for model solutions containing a maximum of three profiles, calculated using the four methods of parametrization. The best solution is indicated by a black box*

| 1 | 2 | *3* | *4* | *5* | *7* | *8* | *9* | *10* | *11* | *12* |
| --- | --- | --- | --- | --- | --- | --- | --- | --- | --- | --- |
| **Ʃ**_k_ | # of classes | *LL* | *npar* | *AIC* | *BIC* | *SABIC* | *CAIC* | *BLRT*  *p*-value | Adj.  LMR-LRT  *p*-value | Entropy |
|  | (K) |  |  |  |  |  |  |  |  |  |
| Class-invariant, diagonal | 1 | -1324.93 | 8 | 2665.87 | 2694.07 | 2668.71 | 2702.07 | < .05 | < .001 | 1.00 |
| **Ʃ**_k_ **Ʃ** | 2 | -1207.32 | 16 | 2440.64 | 2486.47 | 2445.26 | 2499.47 | < .05 | < .05 | 0.92 |
|  | 3 | -1198.54 | 24 | 2433.07 | 2496.53 | 2439.47 | 2514.53 |  |  | 0.77 |
| Class-varying, diagonal | 1 | -1324.93 | 8 | 2665.87 | 2694.07 | 2668.71 | 2702.07 | < .05 | < .001 | 1.00 |
| **Ʃ**_k_ | 2 | -1108.50 | 16 | 2251.00 | 2310.93 | 2257.04 | 2327.93 | < .05 | < .001 | 0.87 |
|  | 3 | -1059.75 | 24 | 2171.49 | 2263.15 | 2180.73 | 2289.15 |  |  | 0.89 |
| Class-invariant, unrestricted | 1 | -1217.59 | 14 | 2463.19 | 2512.54 | 2468.16 | 2526.54 | < .05 | < .001 | 1.00 |
| **Ʃ**_k_ **Ʃ** | 2 | -1163.21 | 22 | 2364.41 | 2431.40 | 2371.16 | 2450.40 | < .05 | < .005 | 0.93 |
|  | 3 | -1150.15 | 30 | 2348.31 | 2432.92 | 2356.84 | 2456.92 |  |  | 0.83 |
| Class-varying, unrestricted | 1 | -1217.59 | 14 | 2463.19 | 2512.54 | 2468.16 | 2526.54 | < .05 | < .001 | 1.00 |
| **Ʃ**_k_ | 2 | -1066.46 | 28 | 2190.91 | 2293.15 | 2201.22 | 2322.15 | < .05 | < .001 | 0.85 |
|  | 3 | -1020.87 | 42 | 2129.73 | 2284.85 | 2145.37 | 2328.85 |  |  | 0.87 |

**References**

Bentler, P. M. (1990). Comparative fit indexes in structural models. *Psychological Bulletin, 107*(2), 238. [https://doi.org/10.1037/0033-2909.107.2.238](https://doi.org/10.1037/0033-2909.107.2.238 )

Brown, T. A. (2006). Confirmatory factor analysis for applied research. New York, NY: Guilford Press.

van Buuren, S. & Groothuis-Oudshoorn, K. (2011). mice: Multivariate imputation by chained equations in R. *Journal of Statistical Software*, *45*(3), 1-67. <https://www.jstatsoft.org/v45/i03/> [https://doi.org/10.18637/jss.v045.i03](file:///C:\Users\2109123\Dropbox\AHRC%20Time%20project\Writeups\Beliefs%20paper\ https:\doi.org\10.18637\jss.v045.i03 )

van Buuren, S. (2018). *Flexible imputation of missing data.* CRC press. [https://doi.org/10.1201/9780429492259](https://doi.org/10.1201/9780429492259 )

Byrne, B. M. (1991). The Maslach Burnout Inventory: Validating factorial structure and invariance across intermediate, secondary, and university educators. *Multivariate Behavioral Research, 26*(4), 583-605. <https://doi.org/10.1207/s15327906mbr2604_2>

Child, D. (2006). *The Essentials of Factor Analysis. (3rd ed.).* New York, NY: Continuum International Publishing Group.

Field, A., Miles, J., & Field, Z. (2012). *Discovering Statistics Using R.* Sage publications.

Gorsuch, R. (1983). *Factor analysis.* Hillsdale, NJ: L. Erlbaum Associates.

Haahr, M. (2010). *Random.org: True random number service.*School of Computer Science and Statistics, Trinity College, Dublin, Ireland. Website (<http://www.random.org>).

Hooper, D., Coughlan, J., & Mullen, M. R. (2008). Evaluating model fit: A synthesis of the structural equation modelling literature. *Journal of Business Research Methods, 6*(1*)*, 53-60.

Hu, L., & Bentler, P. M. (1999). Cutoff criteria for fit indexes in covariance structure analysis: Conventional criteria versus new alternatives. *Structural Equation Modeling*, *6*, 1–55.

MacCallum, R. C., Browne, M. W., & Sugawara, H. M. (1996). Power analysis and determination of sample size for covariance structure modelling. *Psychological Methods, 1*, 130-149.

R Core Team. (2018). *R: A Language and Environment for Statistical Computing.* Vienna, Austria. Retrieved from <https://www.R-project.org/>

Revelle, W. (2019). *psych: Procedures for Psychological, Psychometric, and Personality Research*. Northwestern University, Evanston, Illinois. R package version 1.9.12, [https//CRAN.R-project.org/package=psych](https://cran.r-project.org/package=psych).

Steiger, J. H., & Lind, J. M. (1980, June). Statistically based tests for the number of common factors. Paper presented at the meeting of the Psychometric Society, Iowa City, IA

Stevens, J. P. (1992). *Applied multivariate statistics for the social sciences.* Routledge.

Tabachnick, B. G., & Fidell, L. S. (2013). *Using multivariate statistics: International edition.*Pearson. <https://doi.org/10.4236/ijg.2014.55049>

Tucker, L. R., & Lewis, C. (1973). A reliability coefficient for maximum likelihood factor analysis. *Psychometrika, 38*, 1–10.

Akogul, S., & Erisoglu, M. (2017). An approach for determining the number of clusters in a model-based cluster analysis. *Entropy, 19*(9), 452.

Spurk, D., Hirschi, A., Wang, M., Valero, D., & Kauffeld, S. (2020). Latent profile analysis: A review and “how to” guide of its application within vocational behavior research. *Journal of Vocational Behavior*, *120*, 103445. <https://doi.org/10.1016/j.jvb.2020.103445>
